# Mechanistic insights into niosomal epicatechin on nutritional value and functional quality of chicken meat and economics: monitoring color and oxidative stability for extended shelf life during frozen storage

**DOI:** 10.3389/fvets.2026.1960674

**Published:** 2026-10-13

**Authors:** H. S. Al-Khalaifah, Marwa I. Abd El-Hamid, Sara E. Shahin, Aya M. Emam, Amal S. A. El Oksh, Marwa Samir, Nour El-Hoda Khayrat Hammad, Samah A. M. Abou Shehatta, Elham Elsayed Abo-Almagd, Dalia W. A. H. Elged, Dalia Ibrahim Mohamed, Wessam Youssef, Sherief M. Abdel-Raheem, Doaa Ibrahim

**Affiliations:** 1 Environment and Life Sciences Research Center, Kuwait Institute for Scientific Research, Safat, Kuwait; 2 Department of Microbiology, Faculty of Veterinary Medicine, Zagazig University, Zagazig, Egypt; 3 Department of Animal Wealth Development, Veterinary Economics and Farm Management, Faculty of Veterinary Medicine, Zagazig University, Zagazig, Egypt; 4 Food Hygiene Department, Animal Health Research Institute (AHRI), Mansoura Branch, Agriculture Research Center (ARC), Giza, Egypt; 5 Department of Biotechnology, Reference Laboratory for Quality Control of Poultry Production (RLQP), Animal Health Research Institute (AHRI), Agriculture Research Center (ARC), Zagazig, Egypt; 6 Biochemistry Department, Animal Health Research Institute (AHRI), Mansoura Branch, Agriculture Research Center (ARC), Giza, Egypt; 7 Department of Biochemistry, Animal Health Research Institute (AHRI), Mansoura Branch, Agriculture Research Center (ARC), Giza, Egypt; 8 Agricultural Research Center, Department of Food Hygiene, Food Inspection Laboratory Damietta Port, Animal Health Research Institute, Damietta, Egypt; 9 Department of Nutrition, Oncology Center Faculty of Medicine Mansoura University, Mansoura, Egypt; 10 Department of Toxicology and Biochemical, Zagazig Branch Animal Health Research Institute (AHRI) Agricultural Research Centre (ARC), Zagazig, Egypt; 11 Department of Biochemistry, Animal Health Research Institute, Zagazig Branch, Agriculture Research Center, Zagazig, Egypt; 12 Department of Biotechnology, Agriculture Research Center (ARC), Animal Health Research Institute (AHRI), Giza, Egypt; 13 Department of Public Health, College of Veterinary Medicine, King Faisal University, Al Hofuf, Al Ahsa, Saudi Arabia; 14 Department of Nutrition and Clinical Nutrition, Faculty of Veterinary Medicine, Zagazig University, Zagazig, Egypt

**Keywords:** immune defense, meat quality, muscle development, niosomal epicatechin, oxidation stability, performance

## Abstract

**Introduction:**

In the modern poultry industry, nanoencapsulation protects natural antioxidants like epicatechin from harsh conditions, boosting their effectiveness.

**Methods:**

In this study, broilers were fed four diets with 0, 75, 150, and 300 mg/kg niosomal epicatechin (Nio/Epicatechin) to evaluate its boosted effects on performance, immunity, meat quality, antioxidant stability, and lipid peroxidation during frozen storage.

**Results and discussion:**

The highest dose (300 mg/kg) produced the best results: chickens grew faster, converted feed more efficiently, and had higher carcass yields. Muscle growth genes (MyoD, mTOR, MyoG) were turned on, while the growth-limiting gene MSTN was suppressed. Meat quality improved, with lower drip, thaw, and cooking losses, and better pH stability. Profits increased, and feed costs dropped. Fat and cholesterol in breast and thigh meat were significantly reduced, as fat-making genes (FAS and ACC) were downregulated. After 90 days of frozen storage, meat from the 300 mg/kg group had higher antioxidant capacity, more phenolics and flavonoids, and lower levels of harmful oxidation products like malondialdehyde and cholesterol oxides. Antioxidant enzyme genes (SOD-1, catalase, GSH-Px, NQO1, HO-1) were more active, and the meat showed stronger free radical scavenging power. Overall, Nio/Epicatechin supplementation, especially at 300 mg/kg, enhances growth, improves meat nutritional quality, boosts oxidative stability during frozen storage, and extends shelf life, making it a valuable strategy for poultry production.

## 1 Introduction

Optimizing growth performance, feed utilization, and meat quality while improving economic efficiency and reducing synthetic additives remains a central focus in modern poultry production (1–4). Bioactive phytochemicals, especially polyphenolic compounds, have gained increasing attention as natural feed supplements on account of their strong antioxidant, anti-inflammatory, health-promoting, and meat-preserving capacities (5). Recent nutrigenomic studies in poultry suggest that dietary natural antioxidants can modulate redox-sensitive transcription factors, thereby enhancing stress tolerance, maintaining cellular redox equilibrium, and optimizing metabolic processes (5–8). Among these natural compounds (9), epicatechin, a flavan-3-ol abundant in cocoa, tea, and various fruits, stands out as a potential feed additive (10). Epicatechin contains numerous hydroxyl groups attached to its aromatic rings, which provide strong antioxidant activity by donating hydrogen atoms or electrons and helping neutralize reactive oxygen species (10). Its pronounced redox activity, ability to neutralize reactive oxygen species, and regulatory role in gene expression associated with antioxidant defense, metabolism, and immunity make it a valuable candidate for enhancing broiler performance and meat quality attributes (11). It can also chelate transition metal ions and inhibit lipid peroxidation by interrupting free radical chain reactions (12). These mechanisms help maintain redox balance and protect cellular components from oxidative injury. Because oxidative stress is recognized to impair broiler growth rate, feed utilization, and meat stability (13–15), dietary inclusion of epicatechin can strengthen the antioxidant defense system, reduce lipid and protein oxidation, and ultimately enhance physiological performance and meat quality of broilers (10). These positive outcomes also enable broilers to utilize nutrients more efficiently for growth rather than for managing oxidative stress. Likewise, dietary inclusion of natural polyphenol sources, including grape seed or peel extracts, has been reported to elevate endogenous antioxidant enzyme activities (SOD, CAT, and GPx) without adversely affecting feed intake or growth performance (16). Nevertheless, these effects are influenced by factors such as dosage level, duration of feeding, and bird physiological status, emphasizing the need for optimized supplementation strategies.

Meat quality plays a vital role in determining both consumer preference and market value. Chicken meat is susceptible to oxidative degradation owing to its high content of polyunsaturated fatty acids (17, 18). Oxidative deterioration in meat is observed as nutrient and drip losses, discoloration, and limited shelf life (19). Parameters such as ultimate pH, color attributes (L*, a*, b*), cooking loss, and thawing loss are closely linked to the oxidative status and structural integrity of muscle tissues (20). Oxidative deterioration of membrane lipids and proteins can lead to excessive drip and thaw losses, discoloration, and undesirable changes in tenderness and juiciness (5). Supplementation with natural antioxidants such as catechins and other polyphenols, particularly from grape pomace or green tea extracts, has been reported to lower malondialdehyde (MDA) levels, preserve pigment stability, and improve overall meat quality during storage (21, 22).

From an economic perspective, financial assessments of natural feed additives show that polyphenolic compounds can enhance profitability by improving nutrient utilization, oxidative stability, and meat shelf life (23, 24). Consequently, incorporating epicatechin may represent a sustainable and economically beneficial strategy for optimizing both performance and meat quality in modern poultry production systems. Regardless of its promising biological effects, epicatechin’s physicochemical properties restrict its clinical application, as it exhibits low bioavailability, poor water solubility, and a high tendency for enzymatic and chemical degradation in environmental and biological circumstances, which can significantly affect epicatechin stability, resulting in rapid loss of its activity and therapeutic effectiveness (25). Nanotechnology offers innovative approaches to these challenges by encapsulating bioactive molecules within nanocarriers, improving their stability under acidic gastric conditions and permitting compound release in the intestinal environment (26, 27). In broiler chickens, researchers have evaluated several polyphenolic compounds and polyphenol-rich extracts as functional feed additives, either in free or encapsulated form. Dietary epigallocatechin gallate improved meat quality and muscle antioxidant capacity in heat-stressed broilers (10) and restored lipid metabolism while enhancing meat quality in high-fat-fed birds (11). Likewise, grape skin and vinification by-products elevated antioxidant status and oxidative stability of broiler meat (21, 22), and dietary olive leaf phenolic extract improved growth performance, immune function, and meat antioxidant potential during frozen storage (5). More recently, nanoformulation strategies have been applied to enhance the delivery of such bioactives in broilers; magnetite iron nanoparticles combined with quercetin improved performance, antioxidant and immune defense, and controlled *Clostridium perfringens* infection (28), garlic nanohydrogel optimized growth, gastrointestinal integrity, and economic returns (29), and proanthocyanidin-based nanoformulations combined with bacteriophages enhanced resistance to *Salmonella Typhimurium* (30). Cornelian cherry extract has also been shown to modulate antioxidant biomarker expression and improve the oxidative stability of frozen broiler meat (4). To the authors’ knowledge, however, niosomal encapsulation applied specifically to epicatechin delivery in broiler chickens has not been previously investigated, underscoring the novelty of the present study in simultaneously addressing growth performance, meat quality, antioxidant status, and shelf-life outcomes within a single experimental framework. Therefore, the present study was designed to investigate the impact of dietary niosomal epicatechin (Nio/Epicatechin) supplementation on (i) growth performance parameters, (ii) antioxidant capacity and the expression of key oxidative and immune-related genes, (iii) meat quality attributes and (iv) overall economic efficiency in broiler production as illustrated in Supplementary Figure 1. It was hypothesized that Nio/Epicatechin supplementation would alleviate oxidative stress, beneficially modulate redox and metabolic gene expression, enhance meat quality traits, and improve the economic profitability of broiler production systems.

## 2 Materials and methods

### 2.1 Ethical approval

The study protocol adhered to the animal welfare guidelines for scientific research approved by Zagazig University Institutional Animal Care and Use Committee (ZUIACUC/2/F/235/2025).

### 2.2 Niosomal formulated epicatechin preparation and characterization

Epicatechin (≥ 95% purity; Glentham Life Science, UK; Product code GP4379-5 mg) was used as a bioactive additive. The chemical name is (−)-cis-3,3′,4′,5,7-pentahydroxyflavane (C15H14O6). Niosomes and epicatechin-loaded niosomes were prepared using the procedure described in previous studies (31, 32). In brief, niosomes were prepared by the thin-film hydration method using the non-ionic surfactant Span 60 and cholesterol at a molar ratio of 1:1, together with epicatechin at a surfactant-to-drug ratio of 10:1 (w/w), dissolved in a chloroform:methanol solvent system (2:1, v/v). The hydration step was carried out to obtain a dry thin lipid film by vaporizing the solvents under vacuum overnight, then hydrating in phosphate-buffered saline (pH 7.5) for 40 min at 60 °C, and the resultant niosomal suspension was extruded carefully through a polycarbonate membrane of 200 nm pore size for 10 consecutive extrusion cycles. Next, the polycarbonate membrane filter was used to obtain homogeneous niosomes. After that, the prepared sample was diluted with deionized water, and a drop of the diluted sample was placed on a carbon-copper grid and left to evaporate at room temperature. Finally, the prepared epicatechin-loaded niosomes were characterized using a transmission electron microscope (JEM 2100F, Japan) at 200 kV, and zeta potential was measured as outlined in Figure 1.

**Figure 1:**
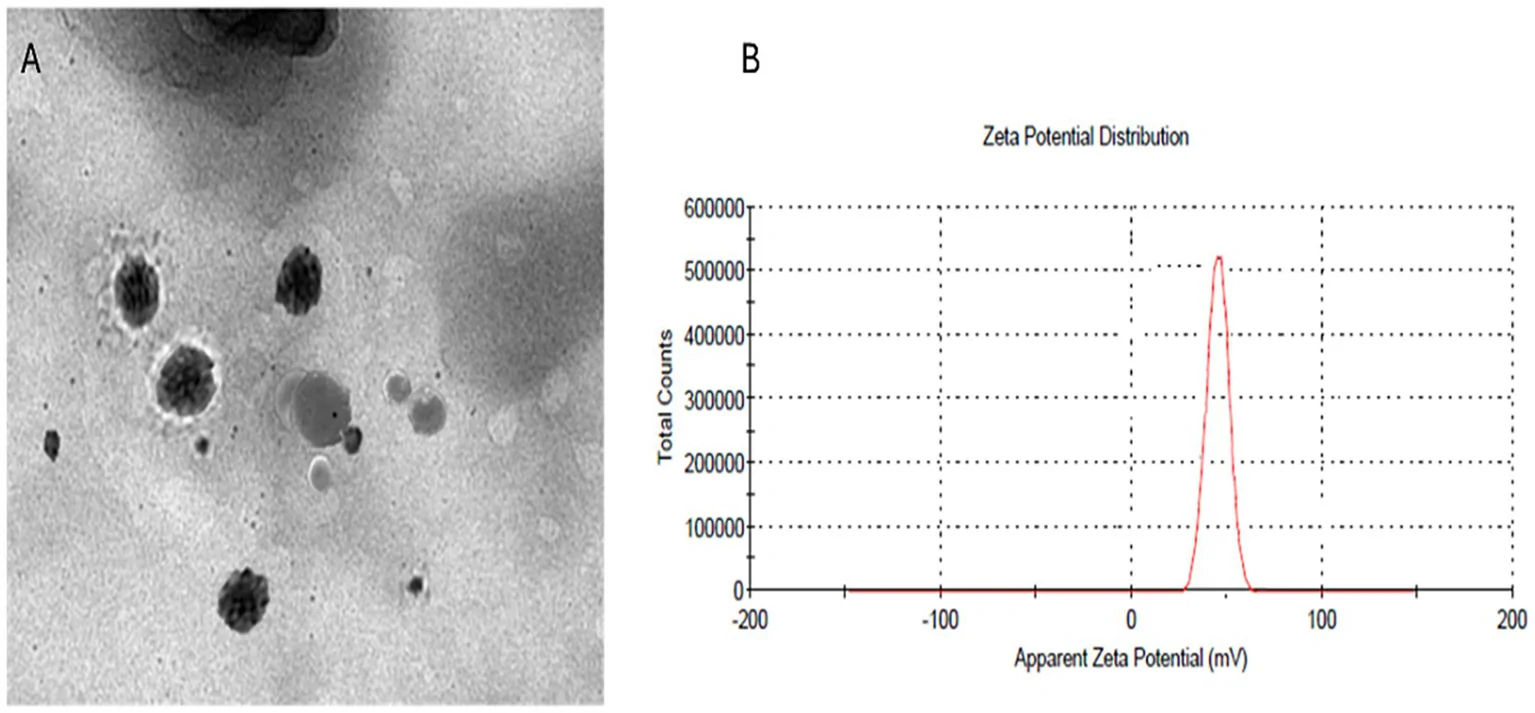
Transmission electron microscopy **(A)** and zeta potential **(B)** analysis of epicatechin-loaded niosomes (Nio/Epicatechin).

### 2.3 Birds, experimental design, and diet

A total of 200 one-day-old male Ross 308 broiler chicks (average initial weight: 44.8 ± 0.4 g) were obtained from a commercial hatchery. Birds were randomly allocated into four dietary treatments, each with five replicates (10 chicks per replicate; 50 chicks per treatment). All birds were reared in floor pens under standard management practices, including temperature control, ventilation, lighting program, and vaccination schedule in accordance with the Ross 308 Management Guide (33). Ambient house temperature was maintained at approximately 33 ± 1 °C during the first week of age and gradually reduced by approximately 3 °C per week to a final temperature of 22 ± 1 °C by day 35; relative humidity was kept between 50–60%, continuous mechanical ventilation was provided, and birds were maintained under a 23 h light: 1 h dark lighting program throughout the trial. Feed and water were provided ad libitum throughout the 38-day experimental period. Throughout the starter (1–10 days), grower (11–20 days), and finisher (21–35 days) phases, birds were fed antibiotic- and coccidiostat-free mash diets formulated to meet the nutritional specifications for Ross broilers (34), as described in Table 1. Crude fiber, ether extract, crude protein, and moisture content of all feed ingredients and experimental diets were determined using the standard procedures of the Association of Official Analytical Chemists (35). Experimental diets were supplemented with epicatechin-loaded niosomes at inclusion levels of 0 mg/kg diet (Control), 75 mg/kg diet (Nio/Epicatechin75), 150 mg/kg diet (Nio/Epicatechin150), and 300 mg/kg diet (Nio/Epicatechin300). The stated inclusion levels of 75, 150, and 300 mg/kg diet refer to the amount of epicatechin itself, back-calculated from the measured entrapment efficiency of the niosomal formulation, rather than to the total mass of the niosomal carrier material.

**Table 1:** The basal diet’s ingredients and nutrient profile.

Ingredient, %	Starter (1–10 days)	Grower (11–20 days)	Finisher (21–35 days)
Yellow corn	58.80	62.00	65.80
Soybean meal, 48%	34.50	30.30	25.50
Soybean oil	1.90	3.00	4.00
Calcium carbonate	1.20	1.20	1.20
Calcium diphasic phosphate	1.50	1.50	1.50
Common salt	0.30	0.30	0.3
DL-Methionine, 99%	0.25	0.2	0.2
L-Lysine HCL, 78%	0.35	0.3	0.3
Premix*	0.90	0.90	0.9
Choline chloride	0.20	0.20	0.20
Anti-mycotoxin	0.10	0.10	0.10
Calculated composition			
Metabolizable energy (Kcal/Kg)	3,106	3,103	3,200
Crude protein, %	23.01	21.5	19.50
Ether extract, %	4.33	5.6	6.62
Crude fiber, %	2.63	2.56	2.46
Methionine, %	0.58	0.51	0.49
Lysine, %	1.45	1.29	1.16
Available phosphorus, %	0.53	0.50	0.48
Calcium, %	1.20	1.19	1.17

^*^Vitamin premix supplied per kilogram of regime: retinol, 12.000 IU; tocopheryl acetate, 80 mg; cholecalciferol, 7,000 IU; menadione, 2.9 mg; mgthiamine, 4 mg; pantothenate, 7 mg; riboflavin, 12 mg; niacin, 53 mg; folate, 3.4 mg; pyridoxine, 6 mg; biotin, 330 μg; cyanocobalamine, 18 μg; Mn (sulphate and oxide), Fe (sulphate), 35 mg; Cu (sulphate), 0.3 mg; I (iodide), 1.20 mg; Zn (sulphate and oxide) and 125 mg; Se (selenate), 16 mg.

### 2.4 Sample harvesting assays

At 38 days of age, five birds per replicate were randomly selected and humanely euthanized by cervical dislocation. Following dissection, breast and thigh meat specimens, abdominal fat, and intestinal tissues were collected to assess the effectiveness of epicatechin-loaded niosomes on nutritional and meat quality, as well as intestinal defense mechanisms and muscle development. Meat tissues were stored at - 20 °C for further biochemical assays, whereas intestinal and muscle tissues were snap-frozen at - 80 °C for molecular analysis.

### 2.5 Productive performance outcomes and cost–benefit assessment

Feed consumption and average body weight (BW) throughout the rearing periods (starter, grower and finisher) were estimated and adjusted to the actual number of live birds present in each replicate at each phase of the trial. Accordingly, body weight gain (BWG) and feed conversion ratio (FCR) were calculated over the whole rearing trial (15, 28, 36, 37). To evaluate the profitability of the experimental diet, the following factors were estimated; total variable cost (TVC) represented as feed cost per kilogram was determined by multiplying the amount of consumed feed (in kilograms) by the cost of feed per kilogram, total cost of production (TC) was estimated by multiplying the cost per kilogram of weight gain by the mean total weight gain, total revenue (TR) was calculated by multiplying the price of meat per kilogram by the mean total weight gain, benefit–cost ratio (BCR) was calculated as the ratio of total returns to total costs and profitability index (PI) was obtained by dividing net profit by total returns, where net profit (NP) is the total returns minus total costs.

### 2.6 Physical and organoleptic properties of chicken breast meat

#### 2.6.1 pH measurement

The pH of meat was measured at 0.5, 12, and 24 h postmortem using a portable pH meter (HI99161, Hanna Instruments, Italy) equipped with a stainless-steel penetration electrode. The device was calibrated with pH 4.0 and 7.0 buffer solutions before use. Each sample was assessed at three different points, and the mean value was documented.

#### 2.6.2 Color measurement

The surface color of cross-sectional breast meat samples was evaluated at 24 h postmortem using a Minolta CR-410 colorimeter (Konica Minolta, Japan) standardized with a white calibration plate. Measurements were expressed in the CIE color system as L (lightness), a (redness), and b (yellowness).

#### 2.6.3 Drip loss

After dressing and evisceration, drip loss was measured as described previously (38). Standardized meat samples (100 g of about 3 cm thickness) were trimmed, weighed (initial weight), suspended in sealed polyethylene bags, and stored at 4 °C for 8 h. Samples were reweighed after blotting excess moisture, and the drip loss % was calculated as follows:

$$\text{Drip loss}\;(\%)=\frac{\text{Initial weight}-\text{Final weight}}{\text{Initial weight}}\times 100$$

#### 2.6.4 Thaw loss

Thaw loss was determined according to previous studies (39, 40). Frozen samples were weighed, thawed at 4 °C for 24 h, blotted, and then reweighed.

$$\text{Thaw loss}\;(\%)=\frac{\text{Frozen weight}-\text{Thawed weight}}{\text{Frozen weight}}\times 100$$

#### 2.6.5 Cooking loss

Cooking loss was assessed as described previously (41). Meat samples were sealed in heat-resistant bags and cooked in a water bath at 85 °C before the heat was applied. Meat was then boiled until an internal temperature of 78 °C was reached (approximately 20 min). After cooling for 15 min under running water, samples were blotted and reweighed to calculate the cooking loss % as follows:

$$\text{Cooking loss}\;(\%)=\frac{\text{Initial}\;\mathrm{raw}\;\text{weight}-\text{cooked weight}}{\text{Initial}\;\mathrm{raw}\;\text{weight}}\times 100$$

#### 2.6.6 Organoleptic evaluation

Breast meat samples were examined for color, odor, taste, and texture as previously described (42). Normal samples exhibited a pinkish-white color, firm texture, and fresh odor, whereas abnormal samples showed discoloration, rancid odor, or off-flavor.

### 2.7 Dressing yield and proximate composition of breast and thigh meat

Carcass traits, including dressing yield, breast and thigh meat weight, and abdominal fat percentages, were recorded at the end of the trial. Dry matter, crude protein, and fat contents were determined according to standard AOAC procedures (35); dry matter by oven-drying to constant weight at 105 °C using a forced-air drying oven (Memmert UF55, Germany); crude protein by the Kjeldahl method using a Kjeltec digestion and distillation system (Kjeltec 8,400, FOSS, Denmark); and crude fat by Soxhlet extraction using a Soxtec extraction system (Soxtec 8,000, FOSS, Denmark). Total cholesterol was quantified using the Folch method (chloroform: methanol, 2:1 v/v) for lipid extraction, followed by gas chromatography (GC) with a flame ionization detector (FID), as outlined elsewhere (43). Chromatographic separation was performed on a fused-silica capillary column (DB-5, 30 m × 0.25 mm i.d. × 0.25 μm film thickness; Agilent J&W, USA), with nitrogen as the carrier gas at a flow rate of 1.0 mL/min. The oven temperature program began at 150 °C (held for 1 min), increased at 10 °C/min to 280 °C and was held for 10 min, with injector and detector temperatures maintained at 280 °C and 300 °C, respectively, using a split ratio of 1:20. The results were presented as mg per 100 g of fresh tissue.

### 2.8 Lipid and cholesterol oxidation markers

The following markers of lipid and cholesterol oxidation were determined in breast meat over 30 and 90 days of frozen storage. Lipid oxidation in stored breast meat was assessed using the thiobarbituric acid–reactive substances (TBARS) assay at 30 and 90 days of storage (−20 °C), following the procedures outlined previously (44, 45). Briefly, extraction of MDA from 5 g of the meat samples was performed using a solution of trichloroacetic acid (5%); a solution of thiobarbituric acid (TBA, 40 mM) was added, and the sample was placed in a water bath for 20 min at 93 °C until complete color development. Finally, MDA was quantified by spectrophotometry (Varian Cary 50, Palo Alto, California) at 532 nm, and results were expressed as μmol MDA/kg tissue.

Total volatile aldehydes were measured using SPME-GC/MS, as detailed elsewhere (46). The chicken meat samples were rinsed with sterile water (5 mL), after which the obtained solution was transferred into a headspace vial (20 mL). Afterward, 9 μL of the 2-octanol solution (0.1638 mg/mL; final concentration 29.425 μg/L) was added as an internal standard. The vial was sealed with a gasket and cap. The samples were then incubated for 20 min at 80 °C. Volatile and semi-volatile substances in the headspace were extracted using a three-phase DVB/CAR/PDMS SPME fiber (50/30 μm; Santa Clara, CA, USA). Before first use, the SPME fiber was conditioned at 250 °C for 1 h. The fiber was then exposed to the vial headspace and allowed to extract analytes at 85 °C for 30 min, followed by thermal desorption in the GC injection port at 250 °C for 5 min. All samples were analyzed in triplicate, and the results were expressed as μmol/kg tissue.

For the determination of cholesterol oxidation products, 250 mg of total lipids were saponified using an NH2-SPE cartridge (Sigma-Aldrich, St. Louis, USA) with an internal standard of 19-hydroxycholesterol (Steraloids, Newport, USA) (47). Elution was performed sequentially with three solvent systems: n-hexane:ethyl acetate (95:5, v/v), n-hexane:ethyl acetate (9:1, v/v), and acetone. Cholesterol oxidation products were derivatized by silylation using a blend of pyridine, hexamethyldisilazane, and trimethylchlorosilane (5:2:1, v/v/v). The resulting silylated derivatives were analyzed via a GC-FID system (GC 2000 Plus, Shimadzu, Columbia, USA). Results were conveyed as mg/kg of tissue. Under the chromatographic conditions applied, 7-ketocholesterol was resolved and quantified, whereas total cholesterol oxidation products were quantified as the sum of all oxysterol peaks resolved in the chromatogram relative to the 19-hydroxycholesterol internal standard.

### 2.9 Antioxidant status of meat samples

#### 2.9.1 Free radical scavenging capability

The free radical scavenging capability of the meat samples was evaluated using the 2,2-diphenyl-1-picrylhydrazyl (DPPH) assay as previously described (48). Briefly, the samples were homogenized in distilled water and centrifuged to obtain the supernatant. The supernatant was then mixed with DPPH radical solution and ethanol, then incubated in the dark for 10 min. Absorbance was measured at 517 nm. The DPPH radical scavenging activity was defined as μM per gram of fresh (moist) meat tissue.

#### 2.9.2 Antioxidant stability

The overall antioxidant activity of the breast meat was determined using an adapted Trolox-equivalent antioxidant capability assay (48). Briefly, the 2,2′-Azino-bis(3-ethylbenzothiazoline-6-sulfonic acid) (ABTS) + radical cation was produced by mixing ABTS (14 mM) with an equal volume of potassium persulfate (4.9 mM), Sigma-Aldrich, USA. The reaction was incubated in the dark at room temperature for 12–16 h. Afterward, the ABTS+ solution (1 mL) was combined with the meat homogenate (10 μL), mixed thoroughly, and the absorbance was then measured after 60 s at 734 nm. The antioxidant capacity was expressed as Trolox equivalents based on the absorbance reading.

#### 2.9.3 Assessment of total antioxidant capacity and total flavonoid and phenolic contents

Total antioxidant capacity (T-AOC) was measured using the colorimetric method provided by the manufacturer (Sigma Co., Cat. No. MAK187). Total flavonoid content was estimated following the method outlined previously (49). In summary, a mixture of 1 mL double-distilled water (Sigma-Aldrich, MO, USA) and 0.25 mL of the meat sample was prepared. To this, 1 M NaOH (0.5 mL), 10% AlCl₃ (0.075 mL) and NaNO₂ (0.075 mL), Oxoid, UK were added sequentially. The volume was then brought up to 2.5 mL with double-distilled water. The absorbance of the resulting solution was measured at 410 nm using a UV–visible spectrophotometer. Quercetin (Sigma-Aldrich, MO, USA) was used as a reference standard to determine the total flavonoid content. Results were expressed as micrograms of quercetin equivalents (QE) per gram of tissue.

The total phenolic content of the breast meat samples was evaluated using the protocol described earlier (50). To 100 μL of the homogenized meat sample, 50% distilled water (2.5 mL), 95% ethanol (500 μL) and Folin–Ciocalteu reagent (250 μL), all from Sigma-Aldrich, MO, USA were added. After mixing, the solution was permitted to stand for 5 min. Then, 5% Na₂CO₃ (250 μL), Sigma-Aldrich, MO, USA, was added to the mixture, which was vortexed and kept in the dark for 1 h. The absorbance of the solution was measured at 725 nm using a spectrophotometer. The total phenolic content was calculated as milligrams of gallic acid equivalents per 100 grams of meat.

### 2.10 Gene expression investigation using reverse transcription-quantitative polymerase chain reaction

After the dietary fortification of Nio/Epicatechin, the expression levels of genes regulating antioxidant enzymes [superoxide dismutase-1 (SOD-1), glutathione peroxidase (GSH-Px), catalase, heme oxygenase-1 (HO-1) and NAD(P)H:quinone oxidoreductase 1 (NQO1)], tight junction and immunity [avian β-defensin 1 (AVBD1), cathelicidin-2, zona occludin, junctional adhesion molecule (JAM), interleukin (IL)-4, IL-10 and transforming growth factor-beta (TGF-β)], lipogenesis [fatty acid synthase (FAS) and acetyl-CoA carboxylase (ACC)] and muscle development [myostatin (MSTN), myogenin (MyoG), myogenic differentiation 1 (MyoD), insulin-like growth factor-1 (IGF-1) and mechanistic target of rapamycin (mTOR)] were examined in broilers intestinal, meat and liver samples using the RT-qPCR technique. Total RNA was extracted from the examined samples using the QIAamp RNeasy Mini Kit (Qiagen, Germany), following the manufacturer’s protocol. The purity of extracted RNA was assessed spectrophotometrically through a NanoDrop® ND-1000 (Thermo Scientific, USA). The reverse transcription-quantitative polymerase chain reaction (RT-qPCR) analyses were achieved, in triplicate, on the MX3005P real-time PCR system (Stratagene Co., USA) utilizing the QuantiTect SYBR Green RT-PCR Kit (Qiagen, Germany) and definite primers for the target analyzed genes ensuing the manufacturer’s directions. Gene expression levels were calculated using the comparative cycle threshold (CT) method (2−∆∆Ct approach) (51) with results expressed as fold changes and further standardized to glyceraldehyde 3-phosphate dehydrogenase (GAPDH), an endogenous housekeeping gene. The data were further equated to the calibrator. A melting curve analysis was conducted to ensure the absence of nonspecific amplification. The sequences of the primers used for housekeeping and target genes in the RT-qPCR assays are detailed in Table 2.

**Table 2:** Primers’ sequences used for reverse transcription-quantitative polymerase chain reaction analysis.

Specificity/target gene	Primer sequence (5′-3′)	Accession No
Antioxidant enzymes		
NQO1	F: TCGCCGAGCAGAAGAAGATTGAAG R: CGGTGGTGAGTGACAGCATGG	NM_001277620.1
HO-1	F: AAGAGCCAGGAGAACGGTCA R: AAGAGCCAGGAGAACGGTCA	NM_205344
GSH-Px	F: AACCAATTCGGGCACCAG R: CCGTTCACCTCGCACTTCTC	HM590226
SOD-1	F: GGCAATGTGACTGCAAAGGG R: CCCCTCTACCCAGGTCATCA	NM_205064.1
Catalase	F: GGGGAGCTGTTTACTGCAAG R: GGGGAGCTGTTTACTGCAAG	NM_001031215.2
Muscle development		
mTOR	F: CATGTCAGGCACTGTGTCTATTCTC R: CTTTCGCCCTTGTTTCTTCACT	XM_417614.5
MyoG	F: GGAGAAGCGGAGGCTGAAG R: GCAGAGTGCTGCGTTTCAGA	NM_204184.1
MyoD	F: CAGCAGCTACTACACGGAATCA R: GGAAATCCTCTCCACAATGCTT	NM_204214.2
MSTN	F: ATGCAGATCGCGGTTGATC R: GCGTTCTCTGTGGGCTGACT	NM_001001461.1
IGF-1	F: GCTGCCGGCCCAGAA R: ACGAACTGAAGAGCATCAACCA	NM_001004384.2
Tight junction and immunity		
Zona occludin	F-ACGGCAAAGCCAACATCTAC R- ATCCGCCACGTTCTTCAC	XM_031604121.1
JAM-2	F-AGACAG GAACAGGCAGTGCT R- TCCAATCCCATTTGA GGCTA	XM_031556661.1
AVBD1	F: AGCCTGTCTGCCTGGAGTAG R: GATGAGGAGAGGCTTCATGG	XM017337690.1
Cathelicidins-2	F-AGGAGAATGGGGTCATCAGG R-GGATCTTTCTCAGGAAGCGG	NM_001024830.3
IL-10	F: GCTGAGGGTGAAGTTTGAGG R: AGACTGGCAGCCAAAGGTC	XM_025143715.1
IL-4	F: TTATGCAAAGCCTCCACAATTG R: GTGGGACATGGTGCCTTGAG	ENSGALG00000006827
TGF-β	F: TATCATCACCAGGACAGCGT R: ACCTTGTGGCTTAGGGTCTG	NM_001031045.3
Lipogenesis		
ACC	TTGTGGCACAGAAGAGGGAA GTTGGCACATGGAATGGCAG 161	NM_205505.1
FAS	F: AGAGGCTTTGAAGCTCGGAC R: GGTGCCTGAATACTTGGGCT	NM_205155.2
Housekeeping gene		
GAPDH	F: GGTGGTGCTAAGCGTGTTA R: CCCTCCACAATGCCAA	NM205518

NQO1, NAD(P)H:quinone oxidoreductase 1; HO-1, heme oxygenase-1; GSH-Px, glutathione peroxidase; SOD-1, superoxide dismutase 1; mTOR, mechanistic target of rapamycin; MyoG, myogenin; MyoD, myogenic determination factor 1; MSTN, myostatin, IGF-1, insulin-like growth factor-1, JAM, junctional adhesion molecule; AVBD1, avian β-defensin 1; IL-10, nterleukin-10; IL-4, interleukin- 4; TGF-β, transforming growth factor-beta; ACC, acetyl-CoA carboxylase; FAS, fatty acid synthase; GAPDH, glyceraldehyde 3-phosphate dehydrogenase.

### 2.11 Statistical analysis

The results were statistically analyzed using SPSS Inc. software version 20 (IBM Corp., NY, USA). Levene and Shapiro–Wilk tests were used to define the experimental groups’ homogeneity and normality, respectively. Furthermore, to define the efficacy of dietary Nio/Epicatechin inclusion, a one-way ANOVA test and Tukey’s test were employed. The standard error of the mean (SEM) was utilized to express the findings’ differences at a significant value of *p* < 0.05. Graphical representation was done using GraphPad Prism Version 8 (CA, USA). The dose–response pattern was further examined using orthogonal polynomial contrasts, which partitioned the treatment sum of squares into linear and quadratic components. Each contrast was tested against the pooled error mean square, and the resulting probabilities (p-linear and p-quadratic) are reported alongside the ANOVA *p*-value. Where a significant quadratic component was detected, the response was described by the second-order model Y = b0 + b1X + b2X2, in which X is the dietary Nio/Epicatechin level (mg/kg diet), and the level associated with the optimal response was estimated from the vertex of the fitted curve (X = −b1/2b2). The vertex is the dietary level at which the fitted second-order model predicts a maximum or a minimum response. It is a model-derived estimate obtained from the regression equation and does not correspond to any of the four inclusion levels actually fed (0, 75, 150 and 300 mg/kg diet). Each estimated vertex is therefore reported together with its position relative to the experimental range: vertices falling between 0 and 300 mg/kg diet are interpolated predictions inside the range tested, whereas vertices exceeding 300 mg/kg diet are extrapolations beyond the highest level evaluated and were not experimentally validated. Where the quadratic term was not significant, the response was described as a linear trend over the range tested and no vertex was estimated.

## 3 Results

### 3.1 Performance outcomes related to growth parameters and cost–benefit indicators

As displayed in Table 3, no significant differences (*p* > 0.05) were detected in the initial body weight among various experimental groups, indicating homogeneity at the beginning of the trial. Starter body weight and weight gain were significantly (*p* < 0.05) higher in the Nio/Epicatechin300 group than in the control group. Regarding the overall outcomes of the grower stage, dietary inclusion of epicatechin-loaded niosomes at the dose of 300 mg/kg diet markedly enhanced (*p* < 0.05) feed utilization efficiency and growth rate relative to the control group, exhibiting maximized growth performance attributes. Notably, feed intake during the grower stage was significantly (*p* < 0.05) reduced in the Nio/Epicatechin300 group compared with the control group. This reduction in feed consumption went along with a significant improvement in FCR with Nio/Epicatechin300 group showing the lowest FCR value. During the finisher stage, Nio/Epicatechin300 treatment continued to significantly (*p* < 0.05) outperform the control group in terms of productive performance. Feed intake during the finisher stage was significantly lower in Nio/Epicatechin300 group than in the control one resulting in a pronounced improvement in feed conversion ratio in this phase. Overall, total body weight, total gain, total feed intake and overall FCR further confirmed the superiority of the Nio/Epicatechin300 treatment over the control group with significantly (*p* < 0.05) higher growth indices and lower overall FCR values. Additionally, orthogonal polynomial contrasts confirmed that the productive response to dietary Nio/Epicatechin was dose-dependent (Table 3). Over the entire rearing period, final body weight, total body weight gain, total feed intake, and overall FCR all changed linearly with increasing inclusion level (p-linear < 0.001), while a concurrent significant quadratic component (p-quadratic ≤ 0.005) indicated that the rate of improvement progressively diminished toward the upper end of the range tested. The fitted quadratic model for total body weight gain gave a vertex at approximately 247 mg/kg diet. This value is a model-derived predicted optimum that lies inside the experimental range (0–300 mg/kg diet); it is an interpolated estimate and was not itself one of the inclusion levels fed. For overall FCR, no interior vertex was identified, because the response continued to improve across the whole range examined; accordingly, 300 mg/kg diet — the highest level actually tested — gave the best feed conversion among the levels evaluated and represents an experimentally observed rather than a model-predicted optimum. During the starter phase, body weight responded linearly only (p-linear = 0.047; p-quadratic = 0.271), consistent with the shorter exposure period, whereas both linear and quadratic components were significant throughout the grower and finisher phases (*p* ≤ 0.029).

**Table 3:** Growth performance interrelated parameters of broiler chickens in relation to feeding on diets enriched with varying levels of niosomal-formulated epicatechin.

Period (Parameter)	Experimental group				*p* value	SEM	P-linear	P-quadratic
	Control	Nio/Epicatechin 75	Nio/Epicatechin 150	Nio/Epicatechin 300				
Starter (1–10 days)								
Initial BW	45.00	45.00	44.60	44.60	0.06	1.56	0. 28	0.214
BW g/bird	366.72^c^	399.76^b^	399.88^b^	409.98^a^	0.04	11.270	0.047	0.271
BWG g/bird	321.72^c^	354.76^b^	355.28^b^	365.38^a^	0.02	12.885	0.028	0.08
FI g/bird	423.18^a^	416.10^b^	403.84^c^	395.22^d^	0.01	16.828	0.02	0.04
FCR	1.3154^a^	1.1729^b^	1.1367^c^	1.0817^d^	< 0.001	0.04	0.007	0.008
Grower (11–20 days)								
BW g/bird	1281.00^d^	1368.40^c^	1484.00^b^	1512.60^a^	0.02	20.275	< 0.001	0.005
BWG g/bird	914.20^c^	968.800^b^	1084.200^a^	1102.400^a^	0.01	21.350	< 0.001	0.029
FI g/bird	1395.12^d^	1437.06^c^	1498.08^b^	1462.42^a^	0.01	10.780	< 0.001	< 0.001
FCR	1.524^a^	1.484^b^	1.380^c^	1.326^d^	< 0.001	0.01	< 0.01	< 0.01
Finisher (21–35 days)								
BW g/bird	2443.80^d^	2609.40^c^	2634.00^b^	2687.20^a^	0.02	19.550	0.03	0.04
BWG g/bird	1163.060^b^	1240.640^a^	1149.920^b^	1175.020^b^	0.01	11.7	0.03	0.02
FI g/bird	2219.60^b^	2303.60^a^	2074.20^c^	1979.80^d^	0.01	12.500	0.02	0.03
FCR	1.91^a^	1.86^b^	1.80^c^	1.67^d^	< 0.001	0.01	< 0.01	< 0.01
Entire rearing								
BW g/bird	2443.80^d^	2609.40^c^	2634.00^b^	2687.20^a^	0.01	19.550	0.02	0.04
BWG g/bird	2398.80^d^	2564.40^c^	2589.40^b^	2642.60^a^	0.02	14.43	0.03	0.04
FI g/bird	4038.20^a^	4157.20^ab^	3976.20^c^	3837.20^d^	0.02	24.70	0. 29	0.35
FCR	1.68^a^	1.6212^a^	1.54^b^	1.45^c^	< 0.001	0.01	< 0.01	< 0.01

BW, body weight; BWG, body weight gain; FI, feed intake; FCR, feed conversion ratio; control: chicks fed basal diet without any supplements; Nio/Epicatechin 75, 150 and 300: chicks fed basal diet enriched with epicatechin encapsulated in niosomes (Nio/Epicatechin) at the levels of 75, 150 and 300 mg/kg, respectively. Data are shown as means ± standard error of the mean (SEM). ^a–d^Various superscript letters in a row imply statistical significance (*p* < 0.05).

Regarding the cost–benefit assessment, the Nio/Epicatechin300 treatment group exhibited marked (*p* < 0.05) improvements in the economic performance metrics compared with the control group (Table 4). Notably, TR was significantly (*p* < 0.05) higher in the Nio/Epicatechin300 group ($5.143) than in the control group ($4.677), reflecting enhanced productive output. Moreover, NP was significantly (*p* < 0.05) maximized in the Nio/Epicatechin300 treatment ($2.740) than in the control group, which recorded the lowest NP value ($2.301). Although TVC and TC were slightly higher in the Nio/Epicatechin300 group than in the control group, these increases were offset by the significantly (*p* < 0.05) greater returns achieved. Feed cost per kilogram of production was significantly (*p* < 0.05) reduced with epicatechin-loaded niosomes in a dose-dependent manner, with the lowest values in the Nio/Epicatechin300 group (0.667 $), indicating improved feed utilization efficiency. Consequently, the Nio/Epicatechin300 group showed the highest BCR (2.14), whereas the control recorded the lowest value (1.97). Similarly, PI increased significantly (*p* < 0.05) in a dose-dependent manner after supplementation with epicatechin-loaded niosomes, reaching its maximum peak in the Nio/Epicatechin300 group (0.533%), demonstrating superior economic efficiency.

**Table 4:** Economic metrics of broiler chickens in relation to feeding on diets enriched with varying levels of niosomal-formulated epicatechin.

Parameter	Experimental group				*p*-value	SEM
	Control	Nio/Epicatechin 75	Nio/Epicatechin 150	Nio/Epicatechin 300		
TVC ($)	1.78^c^	1.87^a^	1.79^b^	1.80^b^	< 0.001	0.06
TC ($)	2.38^c^	2.47^a^	2.39^b^	2.40^b^	< 0.001	0.06
TR ($)	4.68^c^	4.99^b^	5.04^b^	5.14^a^	< 0.001	0.02
NP ($)	2.301^d^	2.52^c^	2.652^b^	2.74^a^	< 0.001	0.01
Feed cost/kg	0.74^a^	0.73^b^	0.71^c^	0.67^d^	< 0.001	0.001
BCR	1.97^d^	2.02^c^	2.11^b^	2.14^a^	< 0.001	0.02
PI (%)	0.492^d^	0.505^c^	0.526^b^	0.533^a^	< 0.001	0.001

TVC, total variable cost; TC, total cost; TR, Total revenue; NP, net profit; BCR, benefit–cost ratio; PI, profitability index; control: chicks fed a basal diet without any supplements; Nio/Epicatechin 75, 150 and 300: chicks fed a basal diet enriched with epicatechin encapsulated in niosomes (Nio/Epicatechin) at the levels of 75, 150 and 300 mg/kg, respectively. Data are shown as means ± standard error of the mean (SEM). ^a–d^Various superscript letters in a row imply statistical significance (*p* < 0.05).

### 3.2 Carcass traits and proximate composition of broiler chickens’ breast and thigh muscle

Compared with the control group, dietary supplementation with Nio/Epicatechin at different levels markedly (*p* < 0.05) influenced dressing yield, cholesterol content, and proximate composition (Table 5). Dressing percentage improved significantly (*p* < 0.05) in all Nio/Epicatechin-supplemented groups, with the greatest improvement observed with the Nio/Epicatechin300 inclusion level (77.80%) compared with the control group (74.77%). Birds in the Nio/Epicatechin300-supplemented group displayed higher breast and thigh yield percentages (35.71 and 29.55%, respectively) than those in the control group (33.95 and 28.99%, respectively). Abdominal fat percentage showed an opposite trend, decreasing significantly (*p* < 0.05) with Nio/Epicatechin supplementation. Birds in the Nio/Epicatechin300 group had the lowest abdominal fat (0.99%) compared with the control (1.40%), indicating reduced body fat deposition. Furthermore, breast and thigh cholesterol concentrations were significantly lower in all Nio/Epicatechin-treated groups, unlike the control group. The control birds had the highest cholesterol levels (56.96 and 63.83 mg/100 g for breast and thigh, respectively), while the treated groups showed a marked reduction, suggesting a cholesterol-lowering effect of Nio/Epicatechin. In terms of proximate chemical composition, breast crude protein content was significantly (*p* < 0.05) elevated in all Nio/Epicatechin-supplemented groups and reached its highest values at 150 and 300 mg/kg diet, whereas thigh crude protein was significantly increased only at 300 mg/kg diet. Correspondingly, breast total lipid content was significantly (*p* < 0.05) reduced at 150 and 300 mg/kg diet, while thigh total lipid content differed significantly from the control only at the highest inclusion level. Thigh dry matter increased significantly (*p* < 0.05) in all Nio/Epicatechin-supplemented groups relative to the control, whereas breast dry matter was significantly higher than the control only at 75 mg/kg diet, with values recorded at 150 and 300 mg/kg diet being statistically indistinguishable from the control. Polynomial contrasts indicated that carcass dressing yield increased in a strictly linear manner with increasing Nio/Epicatechin level (p-linear < 0.001; p-quadratic = 0.590; R^2^ = 0.99), showing that this response had not yet plateaued within the range examined. Abdominal fat was described by Y = 1.382–2.242 × 10^−3^X + 3.19 × 10^−6^×^2^ (R^2^ = 0.95), for which the fitted curve reaches its minimum at approximately 351 mg/kg diet; because this vertex lies beyond the range of levels tested (0–300 mg/kg diet), it is a model-derived extrapolation and not an experimentally validated optimum, the lowest abdominal fat percentage actually measured being that of the highest level fed (300 mg/kg diet). Breast crude protein followed Y = 22.67 + 4.66 × 10^−3^X − 7.6 × 10^−6^×^2^ (R^2^ = 0.999), whose vertex corresponds to a predicted maximum at approximately 307 mg/kg diet; this estimate likewise lies marginally beyond the highest level evaluated and is therefore extrapolated, although the fitted curve is essentially flat between 250 and 300 mg/kg diet. Thigh total lipid content declined in a purely linear fashion (p-linear < 0.001; p-quadratic = 0.665; R^2^ = 0.99). Because the quadratic component was not significant for carcass dressing yield or for thigh total lipids, no vertex was estimated for these two traits, and their responses are reported only as linear trends across the range tested.

**Table 5:** Carcass traits and proximate composition of broiler chickens’ breast and thigh muscle in relation to feeding on diets enriched with varying levels of niosomal-formulated epicatechin.

Parameter	Experimental group				*p* value	SEM	P-linear	P-quadratic
	Control	Nio/Epicatechin 75	Nio/Epicatechin 150	Nio/Epicatechin 300				
Carcass dressing yield (%)	74.77^c^	75.20^c^	76.240^b^	77.80^a^	<0.001	0.245	<0.001	0.590
Breast weight (%)	33.95 ^b^	34.05 ^b^	34.150 ^b^	35.71^a^	<0.001	0.020	<0.001	<0.001
Thigh weight (%)	28.99 ^b^	28.95 ^b^	28.650^b^	29.55^a^	<0.001	0.054	<0.001	<0.001
Abdominal fat (%)	1.40^a^	1.182^b^	1.154^b^	0.99^c^	<0.001	0.006	<0.001	<0.001
Breast cholesterol (mg/100 g)	56.96^a^	48.178^b^	48.318^b^	49.03^b^	0.02	1.212	0.002	<0.001
Thigh cholesterol (mg/100 g)	63.83^a^	56.26^b^	55.910^b^	56.91^b^	0.002	0.858	<0.001	<0.001
Proximate composition								
Breast (%)								
Dry matter	25.32^b^	25.518^a^	25.408^ab^	25.42^ab^	<0.001	0.007	<0.001	<0.001
Crude protein	22.67^c^	22.95^b^	23.17^a^	23.27^a^	<0.001	0.012	<0.001	<0.001
Total lipids	4.35^a^	4.246^ab^	4.21^b^	4.19^b^	<0.001	0.002	<0.001	<0.001
Thigh (%)								
Dry matter	26.84^b^	27.19^a^	27.39^a^	27.53^a^	<0.001	0.038	<0.001	<0.001
Crude protein	22.20^b^	22.20^b^	22.31^b^	22.46^a^	<0.001	0.004	<0.001	<0.001
Total lipids	5.85^a^	5.70^a^	5.47^ab^	5.18^b^	< 0.04	0.053	<0.001	0.665

Control: chicks fed a basal diet without supplements; Nio/Epicatechin 75, 150, and 300: chicks fed a basal diet enriched with epicatechin encapsulated in niosomes (Nio/Epicatechin) at the levels of 75, 150, and 300 mg/kg, respectively. Data are shown as means ± standard error of the mean (SEM). ^a–d^Various superscript letters in a row imply statistical significance (*p* < 0.05).

### 3.3 Meat quality

Table 6 showed that dietary inclusion of Nio/Epicatechin significantly affected (*p* < 0.05) the pH values, drip and thaw losses, and meat color parameters of broilers’ breast and thigh muscles compared with the control group. The breast pH values measured at 0.5, 12, and 24 h postmortem increased significantly in all Nio/Epicatechin-supplemented groups relative to the control group. Birds fed diets containing Nio/Epicatechin showed higher pH values at each time point. At 0.5 h post mortem, the three supplemented groups did not differ significantly from one another (6.64–6.67 versus 6.44 in the control), whereas at 12 and 24 h the highest values were recorded in the Nio/Epicatechin300 group (6.46 and 5.83, respectively) compared with the control group (6.06 and 5.62, respectively). This indicated improved buffering capacity and reduced postmortem acidification in the treated groups. Moreover, drip and thaw losses were significantly reduced (*p* < 0.05) in birds fed Nio/Epicatechin300 (0.806 and 4.18%, respectively) compared with the control group (1.016 and 4.64%, respectively). Cooking loss was significantly (*p* < 0.05) affected by dietary treatment: the lowest value was recorded in the Nio/Epicatechin300 group (11.89%), the Nio/Epicatechin150 group (12.36%) did not differ from the control (12.45%), and the Nio/Epicatechin75 group showed a significantly higher cooking loss (13.53%). The reduction achieved at the highest inclusion level suggests enhanced meat juiciness and retention of intracellular fluids. Regarding color parameters, Nio/Epicatechin fortification positively affected breast and thigh color characteristics. The breast lightness (L*) was significantly (*p* < 0.05) decreased in the Nio/Epicatechin75 and Nio/Epicatechin300 groups relative to the control, with an intermediate and statistically indistinguishable value at 150 mg/kg diet, indicating slightly darker meat. The A* (redness) value increased significantly (*p* < 0.05) with increasing Nio/Epicatechin levels, showing the highest redness in the Nio/Epicatechin300 group (12.86) compared with the control (11.23). For thigh color, the lightness (L*) value decreased progressively (*p* < 0.05) with higher Nio/Epicatechin inclusion, from 57.62 in the control to 56.30 in the Nio/Epicatechin300 group, while the A* value increased significantly (*p* < 0.05) in all treated groups compared with the control group, suggesting enhanced color stability. B* (yellowness) values of breast and thigh meat varied only marginally among treatments, and the mean separation test did not resolve any pairwise difference between groups. For the meat quality attributes summarized in Table 6, pH at all three postmortem intervals, drip loss and the lightness and redness of both breast and thigh muscle responded both linearly and quadratically to increasing dietary Nio/Epicatechin (*p* ≤ 0.009), whereas thaw loss decreased in a purely linear manner (p-linear < 0.001; p-quadratic = 0.178) and cooking loss showed both linear and quadratic components (p-linear = 0.009; p-quadratic = 0.044). Drip loss was best described by Y = 1.026–1.589 × 10^−3^X + 2.87 × 10^−6^×^2^ (R^2^ = 0.98), with a predicted minimum at 277 mg/kg diet, a vertex that lies within the range of levels tested and therefore represents an interpolated predicted optimum, and breast redness by Y = 11.10 + 1.053 × 10^−2^X − 1.51 × 10^−5^×^2^ (R^2^ = 0.90), with a predicted maximum at 349 mg/kg diet; since this vertex exceeds the highest level fed (300 mg/kg diet) it is a model-derived extrapolation rather than an experimentally validated optimum, and the highest redness value actually measured was that of the Nio/Epicatechin300 group.

**Table 6:** Physical properties (meat quality) of broiler chicken meat in relation to feeding on diets enriched with varying levels of niosomal-formulated epicatechin.

Parameter	Experimental group				*p* value	SEM	P-linear	P-quadratic
	Control	Nio/Epicatechin 75	Nio/Epicatechin 150	Nio/Epicatechin 300				
pH 0.5 h	6.44^b^	6.67^a^	6.64^a^	6.65^a^	< 0.001	0.001	0.01	0.02
pH 12 h	6.06^b^	6.38^a^	6.43^a^	6.46^a^	< 0.001	0.002	0.01	0.03
pH 24 h	5.62^b^	5.65^b^	5.75^a^	5.83^a^	0.003	0.003	0.009	0.03
Drip loss (%)	1.02^a^	0.94^ab^	0.84^ab^	0.81^b^	< 0.001	0.013	0.01	0.01
Thaw loss (%)	4.64^a^	4.57^a^	4.44a	4.18^b^	0.01	0.024	0.03	0.178
Cooking loss (%)	12.45^b^	13.53^a^	12.36^b^	11.89^b^	< 0.01	0.236	0.009	0.044
Meat color								
Breast								
(L*)	56.45^a^	55.40^b^	55.33^ab^	55.24^b^	< 0.001	0.004	0.01	0.02
(A*)	11.23^d^	11.46^c^	12.60b	12.86^a^	< 0.001	0.004	0.02	0.03
(B*)	9.09	9.07	9.158	9.134	0.07	0.004	0.08	0.99
Thigh								
(L*)	57.62^a^	56.854^b^	56.55c	56.30^d^	0.02	0.007	0.03	0.05
(A*)	14.90^b^	15.36^a^	15.29a	15.35^a^	< 0.001	0.004	0.02	0.03
(B*)	10.21	10.178	10.242	10.24	0.75	0.003	0.85	0.97

L*, lightness color parameter; A*, redness color parameter; B*, yellowness color parameter; control: chicks fed a basal diet without any supplements; Nio/Epicatechin 75, 150, and 300: chicks fed a basal diet enriched with epicatechin encapsulated in niosomes (Nio/Epicatechin) at the levels of 75, 150, and 300 mg/kg, respectively. Data are shown as means ± standard error of the mean (SEM). ^a–d^Various superscript letters in a row imply statistical significance (*p* < 0.05).

### 3.4 Organoleptic evaluation

All examined breast meat samples across the four dietary treatments exhibited a normal pinkish-white color, firm texture, and fresh odor throughout the 90-day frozen storage period, with no rancid odor, off-flavor, or discoloration detected by the trained evaluation panel at either the 30-day or 90-day sampling points. No treatment-related differences in organoleptic classification were observed among the Control, Nio/Epicatechin75, Nio/Epicatechin150, and Nio/Epicatechin300 groups.

### 3.5 Meat antioxidant capacity

Table 7 showed that dietary supplementation with Nio/Epicatechin produced a pronounced (*p* < 0.05) improvement in the antioxidant profile of broiler breast meat, unlike the control group. Total phenolic content increased progressively (*p* < 0.05) with rising Nio/Epicatechin levels. Birds fed Nio/Epicatechin300 recorded the highest total phenolic concentrations at both sampling points (153.20 and 132.60 mg GAE/kg at 30 and 90 days post frozen storage, respectively), whereas the control group showed the lowest values (105.80 and 73.20 mg GAE/kg, respectively). These findings indicated a strong dose-dependent increase in phenolic deposition in the muscle tissue. Similarly, total flavonoid content improved considerably (*p* < 0.05) in all Nio/Epicatechin supplemented groups, unlike the control group. The Nio/Epicatechin300 group exhibited the highest flavonoid values (146.60 and 126.00 mg QE/kg at 30 and 90 days post frozen storage, respectively), while the control one recorded the lowest (94.40 and 66.20 mg QE/kg, respectively). The enhancement in antioxidant constituents was reflected in the T-AOC, which rose sharply (*p* < 0.05) in response to Nio/Epicatechin supplementation. At both measurement times, T-AOC was significantly (*p* < 0.05) higher in the groups treated with Nio/Epicatechin300 fed birds showing the greatest activity (17.70 and 14.92 μmol Trolox eq/g at 30 and 90 days post frozen storage, respectively), compared with the control ones (7.81 and 5.40 μmol Trolox eq/g, respectively). Likewise, DPPH radical-scavenging activity and ABTS antioxidant activity were markedly (*p* < 0.05) higher in all Nio/Epicatechin-supplemented groups than in the control group. The Nio/Epicatechin300 group demonstrated the strongest DPPH scavenging capacity (9.39 and 8.04 μmol Trolox eq/g at 30 and 90 days post frozen storage, respectively) and the highest ABTS values at the same intervals (7.67 and 6.93 μmol Trolox eq/g, respectively), while the control group had the lowest antioxidant activities, indicating its long-lasting effect on meat antioxidant stability. All antioxidant indices increased linearly with dietary Nio/Epicatechin level at both 30 and 90 days of frozen storage (p-linear < 0.001; Table 7). A significant quadratic component was also detected for total phenolic and flavonoid contents, DPPH and ABTS at both sampling points and for T-AOC at 90 days (*p* ≤ 0.016), reflecting a progressive attenuation of the response above 150 mg/kg diet. After 90 days of frozen storage the fitted models were Y = 76.15 + 0.4465X − 8.72 × 10^−4^×^2^ for total phenolic content (R^2^ = 0.94), Y = 3.097 + 3.536 × 10^−2^X − 6.34 × 10^−5^×^2^ for DPPH radical-scavenging activity (R^2^ = 0.99) and Y = 5.306 + 6.137 × 10^−2^X − 9.74 × 10^−5^×^2^ for T-AOC (R^2^ = 0.998), corresponding to estimated vertices of 256, 279 and 315 mg/kg diet, respectively. The vertices for total phenolic content (256 mg/kg diet) and for DPPH radical-scavenging activity (279 mg/kg diet) lie within the experimental range and are interpolated predicted optima, whereas that for T-AOC (315 mg/kg diet) falls beyond the highest level evaluated and is an extrapolated, model-derived estimate; for all three indices, the highest values actually measured were those of the Nio/Epicatechin300 group.

**Table 7:** Oxidation stability of broiler chicken meat over 30 and 90 days of frozen storage periods in relation to feeding on diets enriched with varying levels of niosomal-formulated epicatechin.

Parameter	Experimental group				*p-* value	SEM	*P*-linear	*P*-quadratic
	Control	Nio/Epicatechin 75	Nio/Epicatechin 150	Nio/Epicatechin 300				
30 days of frozen storage								
Total phenolic compounds	105.80^d^	126.60^c^	142.00^b^	153.20^a^	0.003	4.05	< 0.001	0.016
Total flavonoids	94.40^c^	120.00^b^	138.80^a^	146.60^a^	< 0.001	4.85	< 0.001	0.005
DPPH	4.62^d^	6.38^c^	7.82^b^	9.39^a^	0.023	0.19	< 0.001	0.002
ABTS	5.44^c^	6.71^b^	7.42^a^	7.67^a^	< 0.001	0.08	< 0.001	< 0.001
T-AOC	7.81^d^	10.69^c^	14.02^b^	17.70^a^	< 0.001	0.72	< 0.001	0.207
90 days of frozen storage								
Total phenolic compounds	73.20^c^	112.60^b^	117.60^b^	132.60^a^	< 0.001	6.27	< 0.001	0.014
Total flavonoids	66.20^d^	105.60^c^	118.60^b^	126.00^a^	< 0.001	6.77	< 0.001	0.004
DPPH	2.97^d^	5.73^c^	6.72^b^	8.04^a^	0.031	0.19	< 0.001	< 0.001
ABTS	2.68^c^	5.97^b^	6.68^a^	6.93^a^	0.004	0.07	< 0.001	< 0.001
T-AOC	5.396^d^	9.12^c^	12.50^b^	14.92^a^	< 0.001	0.50	< 0.001	0.001

DPPH, 2,2-diphenyl-1-picrylhydrazyl; ABTS, 2,2′-Azino-bis(3-ethylbenzothiazoline-6-sulfonic acid); T-AOC, total antioxidative capacity; control: chicks fed basal diet without any supplements, Nio/Epicatechin 75, 150 and 300: chicks fed basal diet enriched with epicatechin encapsulated in niosomes (Nio/Epicatechin) at the levels of 75, 150 and 300 mg/kg, respectively. Data are shown as means ± standard error of the mean (SEM). ^a–d^Various superscript letters in a row imply statistical significance (*p* < 0.05).

Figure 2 showed that dietary supplementation with Nio/Epicatechin significantly (*p* < 0.05) improved the oxidative stability of broiler breast meat compared with the control group, as reflected by reductions in lipid peroxidation products and cholesterol oxidation derivatives. The concentration of MDA, an indicator of lipid peroxidation, was markedly (*p* < 0.05) lower in all Nio/Epicatechin-treated groups than in the control group. At day 30, MDA levels decreased from 0.51 μmol/kg in the control group to 0.20 μmol/kg in the Nio/Epicatechin300 group. A similar trend was observed after 90 days of storage, where MDA dropped sharply from 0.91 μmol/kg in the control group to 0.30 μmol/kg in the Nio/Epicatechin300 group. These reductions indicate strong antioxidant protection and inhibition of lipid oxidation in Nio/Epicatechin-fed birds. The total volatile aldehydes, another marker of oxidative degradation, were also significantly (*p* < 0.05) reduced by Nio/Epicatechin supplementation. At both 30 and 90 days, treated groups showed lower aldehyde concentrations, with the greatest reduction observed in the Nio/Epicatechin300 group (1.27 and 1.58 μmol/kg, respectively) related with the control one (2.06 and 2.88 μmol/kg, respectively). Similarly, ketocholesterol and total cholesterol oxidation products decreased significantly (*p* < 0.05) in the Nio/Epicatechin-supplemented groups. At 30 days, ketocholesterol declined from 1.83 mg/kg in the control group to 1.19 mg/kg in the Nio/Epicatechin300 group, and after 90 days, from 2.70 to 1.42 mg/kg μmol/kg, respectively. The same pattern was observed for cholesterol oxidation products’ values, which were markedly (*p* < 0.05) reduced in all treated groups at both storage periods.

**Figure 2:**
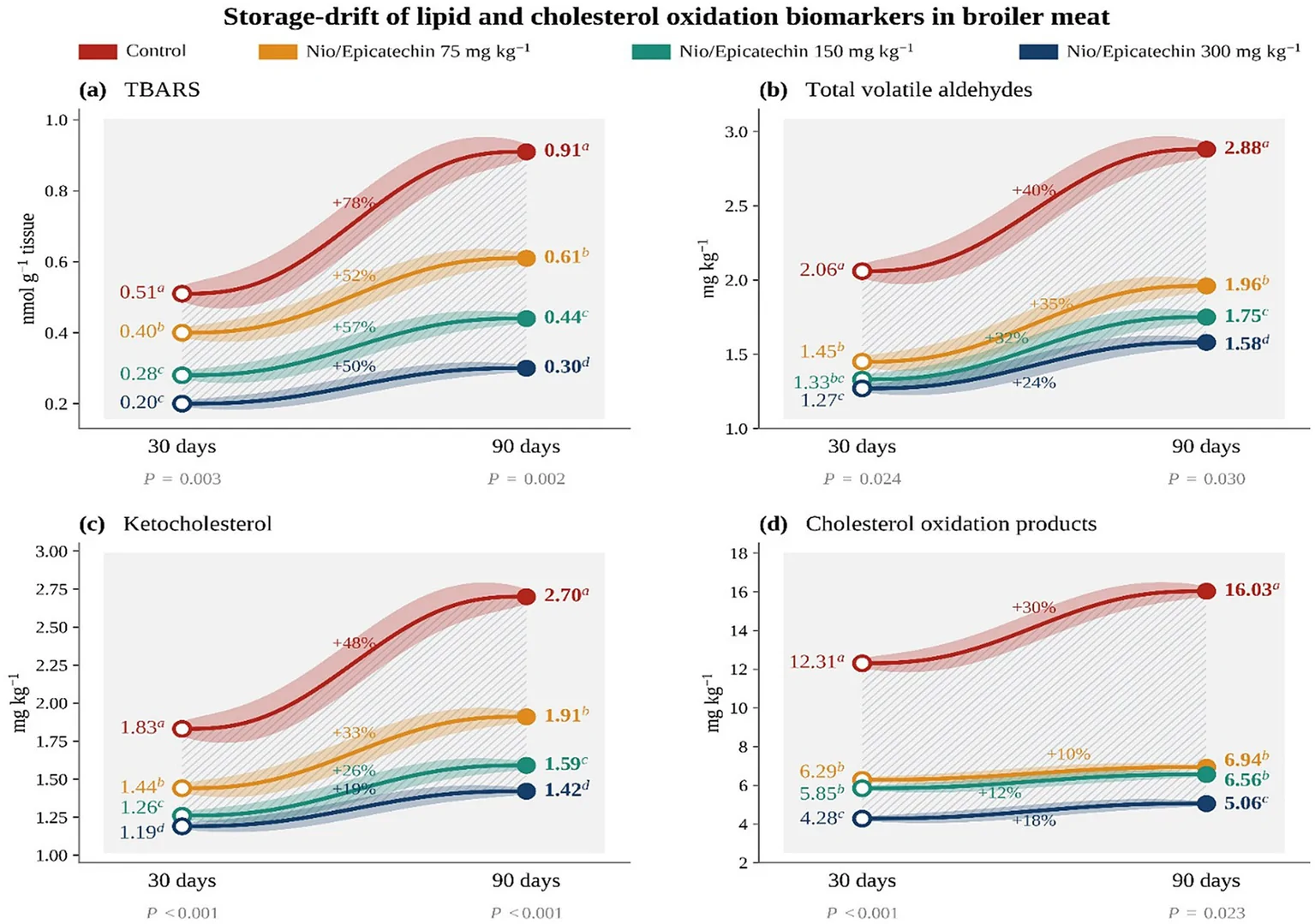
Oxidation of fatty acid-related biomarkers: thiobarbituric acid reactive substances (TBARS) **(a)**, total volatile aldehydes **(b)**, ketocholesterol **(c)**, and cholesterol oxidation products **(d)** of broiler chicken meat over 30- and 90-day frozen storage periods in relation to feeding on diets enriched with varying levels of niosomal-formulated epicatechin. Control: chicks fed a basal diet without supplements; Nio/Epicatechin 75, 150, and 300: chicks fed a basal diet enriched with epicatechin encapsulated in niosomes (Nio/Epicatechin) at the levels of 75, 150, and 300 mg/kg, respectively.

### 3.6 Antioxidant-related genes expression

Figure 3 illustrates the effect of dietary Nio/Epicatechin supplementation on antioxidant enzyme-encoding genes (GSH-Px, catalase and SOD-1) and genes playing a crucial defense mechanism against oxidative stress (HO-1 and NQO1). With increasing levels of Nio/Epicatechin, a significant (*p* < 0.05) dose-dependent increase was observed in the expression levels of GSH-Px and SOD-1 genes. Furthermore, the expression of the catalase gene reached its peak in the group supplemented with Nio/Epicatechin300. The transcriptional levels of HO-1 and NQO1 genes were maximized (1.66- and 1.98-fold changes, respectively) in the group fed Nio/Epicatechin300.

**Figure 3:**
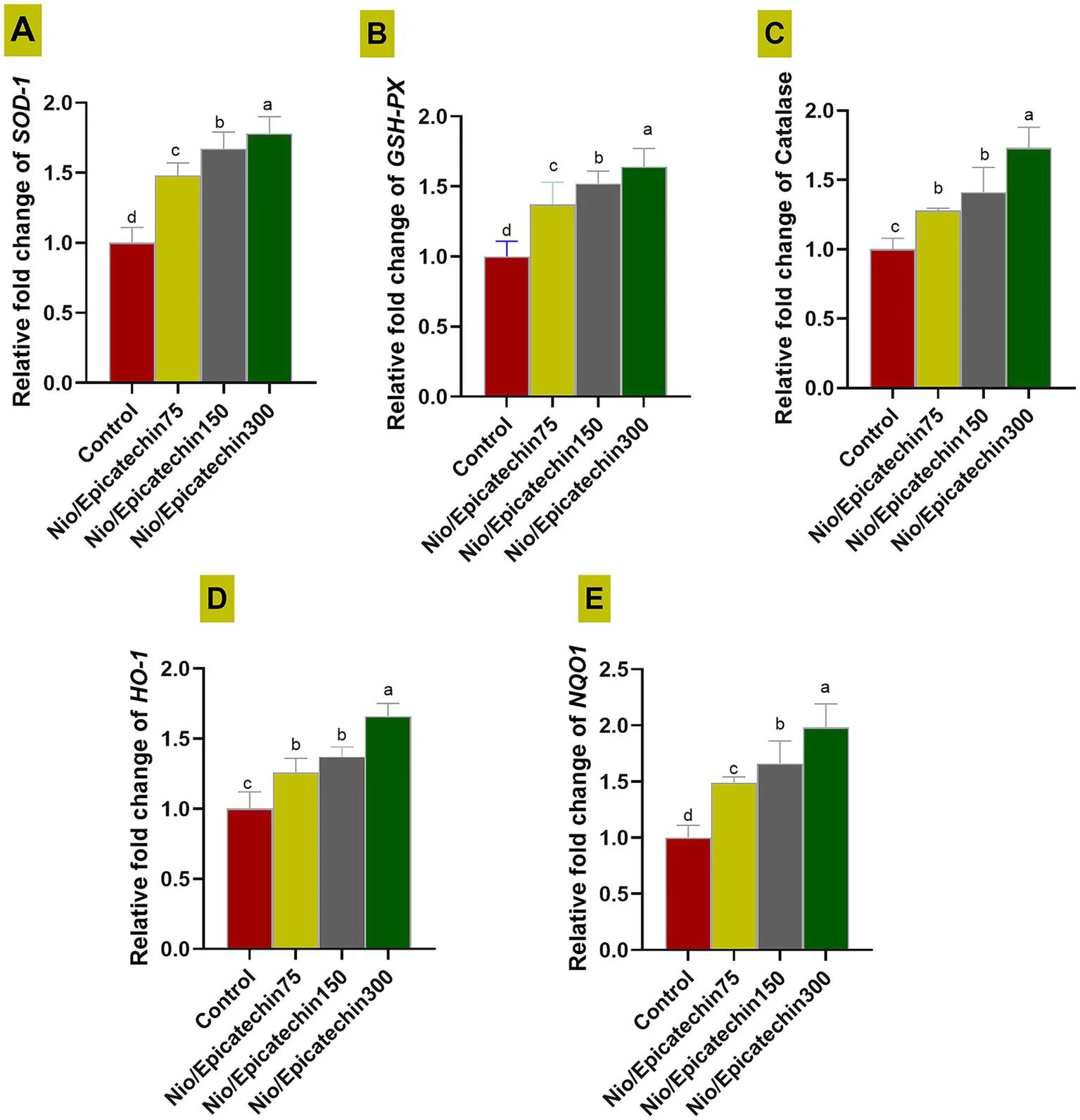
Transcriptional regulation of antioxidant defense-related genes in the breast meat samples of broiler chickens in relation to feeding on diets enriched with varying levels of niosomal-formulated epicatechin (Nio/Epicatechin); superoxide dismutase-1 (SOD-1) **(A)**, glutathione peroxidase (GSH-Px) **(B)**, catalase **(C)**, heme oxygenase-1 (HO-1) **(D)** and NAD(P)H:quinone oxidoreductase 1 (NQO1) **(E)**. Control: chicks fed a basal diet without supplements; Nio/Epicatechin 75, 150, and 300: chicks fed a basal diet enriched with epicatechin encapsulated in niosomes (Nio/Epicatechin) at the levels of 75, 150, and 300 mg/kg, respectively. Results are presented as means ± standard error of the mean (SEM). ^a–d^Values within the same column with different superscripts are substantially different at *p* < 0.05.

### 3.7 Immune defense and tight junction-related gene expression

The mRNA expression of intestinal immune defense and tight junction-encoding genes is shown in Figure 4. A significant (*p* < 0.05) upregulation in the expression of antimicrobial peptide genes, AVBD1 and cathelicidin 2, was observed following dietary supplementation with Nio/Epicatechin compared with the control group. Similarly, the expression levels of tight junction-related genes; zona occludin and JAM, and anti-inflammatory cytokines; IL-4, IL-10 and TGF-β, were significantly (*p* < 0.05) increased and reached their peak in broilers fed diets supplemented with Nio/Epicatechin300.

**Figure 4:**
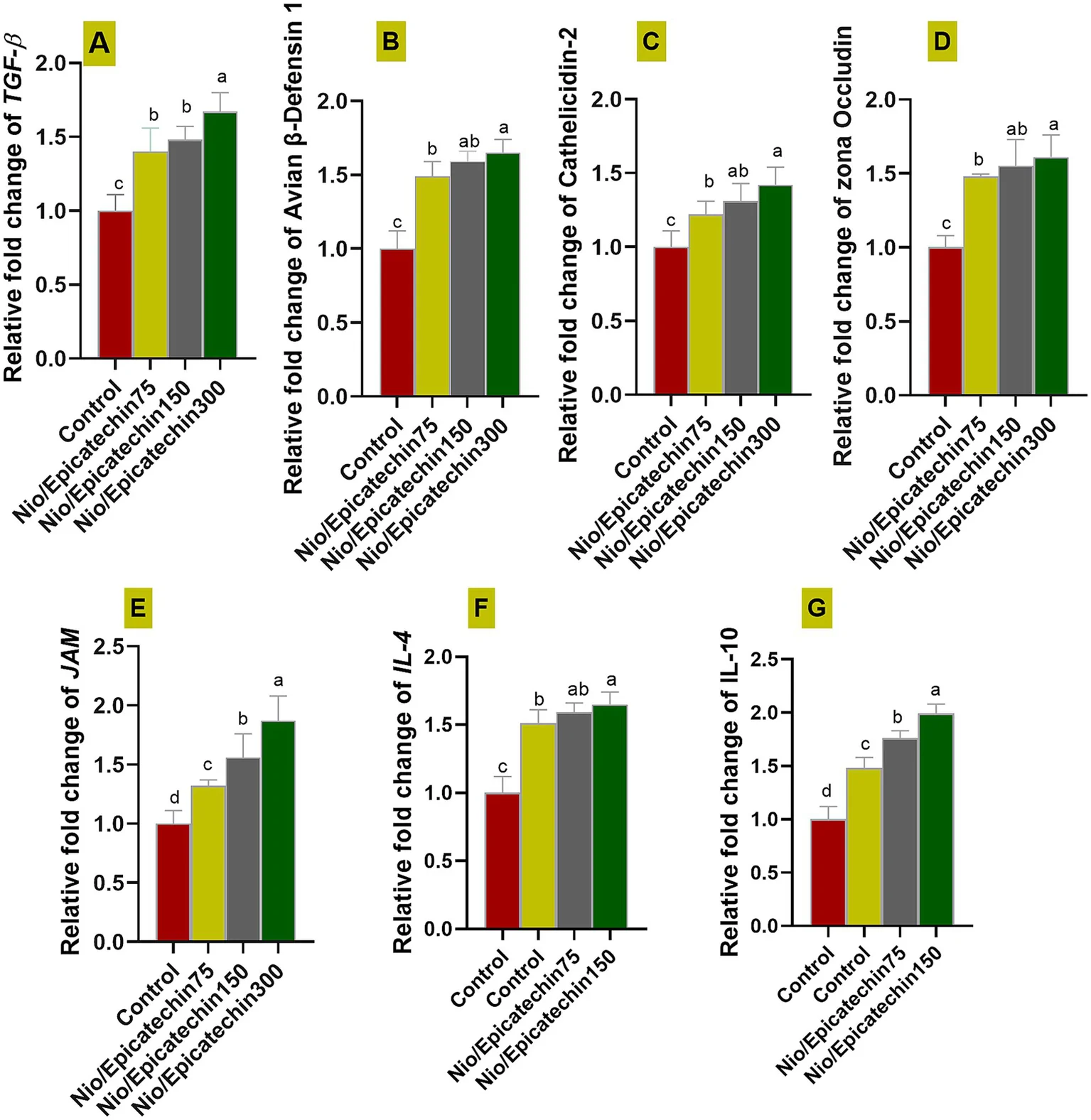
Transcriptional regulation of immune defense and tight junction encoding genes in the intestinal samples of broiler chickens in relation to feeding on diets enriched with varying levels of niosomal-formulated epicatechin (Nio/Epicatechin); transforming growth factor-beta (TGF-β) **(A)**, avian β-defensin 1 (AVBD1) **(B)**, cathelicidin-2 **(C)**, zona occludin **(D)**, junctional adhesion molecule (JAM) **(E)**, interleukin (IL)-4 **(F)** and IL-10 **(G)**. Control: chicks fed a basal diet without supplements; Nio/Epicatechin 75, 150 and 300: chicks fed a basal diet enriched with epicatechin encapsulated in niosomes (Nio/Epicatechin) at the levels of 75, 150, and 300 mg/kg, respectively. Results are presented as means ± standard error of the mean (SEM). ^a–d^Values within the same column with different superscripts are substantially different at *p* < 0.05.

### 3.8 Expression of muscle-building and lipogenesis-interrelated genes

As illustrated in Figure 5, the highest expression levels (*p* < 0.05) of MyoG, MyoD, and IGF-1 genes (1.44-, 1.64-, and 1.51-fold changes, respectively), along with the lowest expression of the MSTN gene (0.12-fold change), were observed in the group supplemented with Nio/Epicatechin300 mg/kg. Moreover, the mRNA expression of the mTOR gene was significantly (*p* < 0.05) upregulated following Nio/Epicatechin supplementation, with more pronounced effects at levels of 150 and 300 mg/kg compared with the control group. Furthermore, broiler chickens fed Nio/Epicatechin at levels of 150 and 300 mg/kg showed the lowest significant (*p* < 0.05) expression of the ACC gene, with reductions of 0.90- and 0.82-fold changes, respectively. Furthermore, the results showed that the expression of the FAS gene was significantly reduced (*p* < 0.05) in all groups fortified with Nio/Epicatechin.

**Figure 5:**
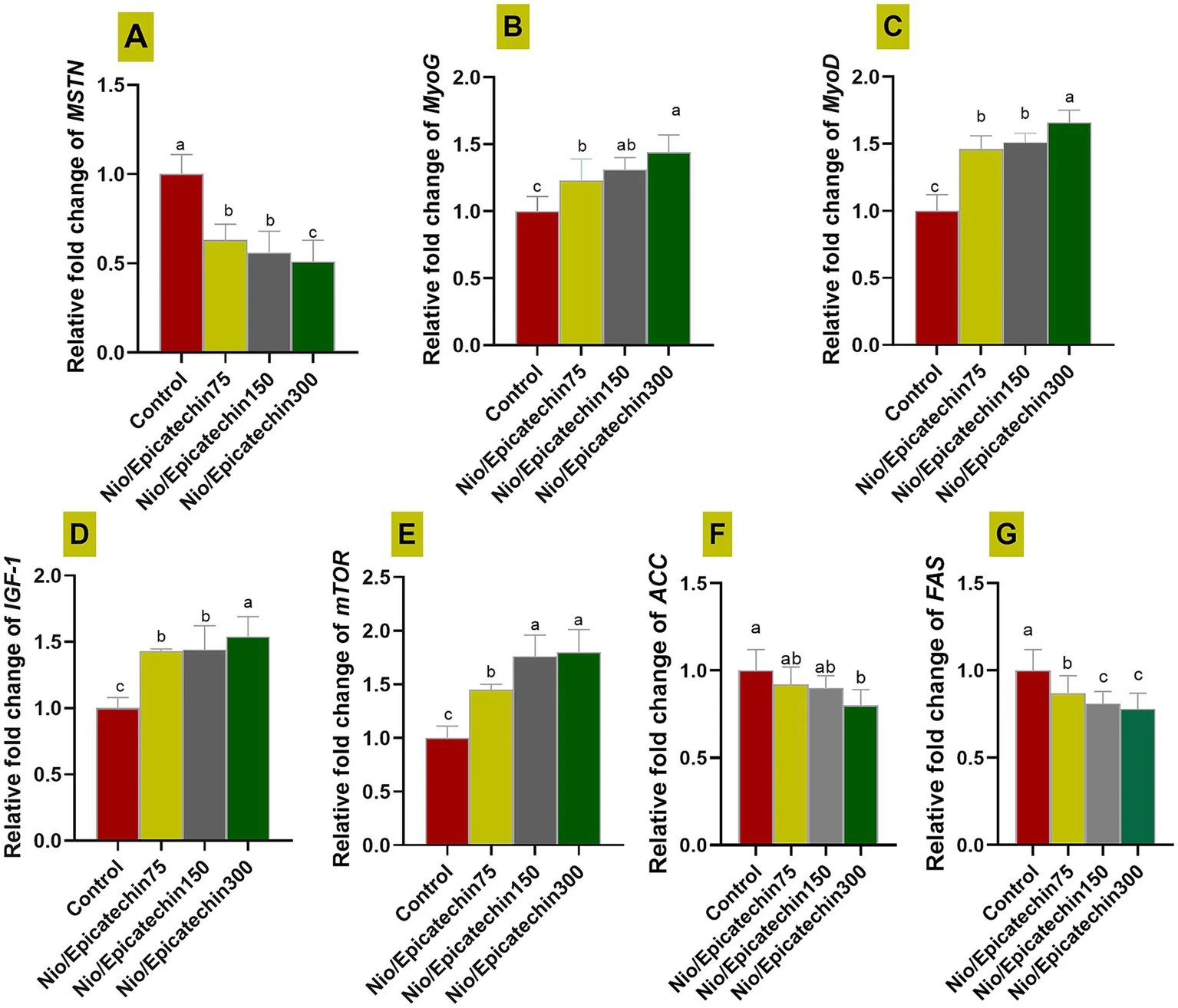
Transcriptional regulation of genes related to muscle development in muscle tissues and lipid metabolism in the liver samples of broiler chickens in relation to feeding on diets enriched with varying levels of niosomal-formulated epicatechin (Nio/Epicatechin); myostatin (MSTN) **(A)**, myogenin (MyoG) **(B)**, myogenic differentiation 1 (MyoD) **(C)**, insulin-like growth factor-1 (IGF-1) **(D)**, mechanistic target of rapamycin (mTOR) **(E)**, acetyl-CoA carboxylase (ACC) **(F)** and fatty acid synthase (FAS) **(G)**. Control: chicks fed a basal diet without any supplements; Nio/Epicatechin 75, 150 and 300: chicks fed a basal diet enriched with epicatechin encapsulated in niosomes (Nio/Epicatechin) at the levels of 75, 150 and 300 mg/kg, respectively. Results are presented as means ± standard error of the mean (SEM). ^a–c^Values within the same column with different superscripts are substantially different at *p* < 0.05.

## 4 Discussion

Intensive broiler production techniques are often associated with increased oxidative stress, particularly in the finisher stage when rapid muscle growth and high metabolic rates are common. Excessive oxidative stress has a negative impact on broilers’ nutrition, metabolism, and muscle protein synthesis (14, 29, 52). Meanwhile, improved antioxidant status allows nutrients and energy to be used preferentially for growth rather than diverted toward oxidative damage repair. In this way, the use of strong scavengers for reactive oxygen species of natural origin, such as epicatechin, has been shown to boost the activity of endogenous antioxidant enzymes, preserving birds’ metabolic efficiency and cellular integrity (53, 54). Despite epicatechin’s biological protective potential, its clinical applications have been hindered by its poor bioavailability, low aqueous solubility, and extreme sensitivity to enzymatic and chemical degradation in biological systems (55). Moreover, its therapeutic effectiveness can be reduced after exposure to harsh environmental conditions involving temperature, light, pH, and the presence of oxygen, affecting its stability (56–58). Therefore, applying prospective strategies that protect epicatechin and improve its stability, delivery, and solubility is vital to expand its applicable antioxidant potential.

In the current study, the improved growth development observed in broilers fed varying levels of biologically active flavanol (epicatechin) in nanoencapsulated niosomes may be attributed to its boosted antioxidant and metabolic regulatory properties. Similarly, polyphenolic compounds such as epicatechin improve intestinal integrity, oxidative state, and nutrient utilization efficiency, all of which enhance the growth performance of broiler chickens (59, 60). Furthermore, the benefits of epicatechin on growth attributes were explained by triggering the activity of digestive enzymes and intestinal structure, which increases absorptive capacity (61). Moreover, the observed improvements in slaughter and dressed weights, along with higher breast and thigh yields in broilers supplemented with Nio/Epicatechin, particularly at higher inclusion levels, occurred alongside its proven benefits for growth performance and carcass development (18). Similarly, dietary supplementation with the free form of epigallocatechin gallate enhanced broilers’ performance, slaughter percentage, breast and leg muscle yield at the level of 400 mg/kg (10). Additionally, epicatechin has been related to increased protein intake and muscular development. Flavonoids can improve muscle development efficiency by altering signaling pathways related to muscle protein synthesis and mitochondrial biogenesis (62). In broilers, improved protein deposition efficiency directly translates into higher body weight gain and improved feed conversion, especially during the finishing phase when breast muscle development predominates (63). Nutrient digestibility and intestinal morphology were not directly measured in the present study; therefore, the mechanism underlying the concurrent improvement in BW and FCR alongside reduced feed intake at 300 mg/kg Nio/Epicatechin remains hypothetical. We propose that improved digestive enzyme activity, gut barrier integrity, and nutrient-partitioning efficiency, consistent with the tight-junction and immune gene expression data reported herein, most plausibly underlie this response; dedicated digestibility trials and intestinal histomorphometric analysis are recommended in future studies to directly confirm this mechanism. These improvements in carcass yield and muscle crude protein content are likely linked to the augmented antioxidant and metabolic modulatory roles of epicatechin when formulated in niosomal form (64). We propose that improved aqueous solubility and a more sustained release profile contributed to enhanced nutrient assimilation and muscle accretion; however, neither solubility nor release kinetics was assessed *in vivo* in the present study, and this interpretation is advanced as a hypothesis supported by the broader nanoencapsulation literature (64) rather than as a mechanism demonstrated experimentally here.

The balance between the myostatin-signaling pathways (negative regulator) and IGF-1/mTOR (positive regulator) assisted proper muscle development (65, 66). In this framework, dietary incorporation of Nio/Epicatechin prompted muscle protein synthesis by upregulating MyoG, MyoD, and mTOR genes and decreased protein degradation through MSTN gene downregulation, which agreed with the improved performance findings. In accordance, epicatechin has also been reported to improve mitochondrial efficiency, mitigate oxidative stress, and modulate protein synthesis pathways such as PI3K/Akt/mTOR, thereby promoting muscle growth while reducing proteolysis. The activation of this signaling cascade leads to muscle fiber hypertrophy and increased protein deposition, coupled with the upregulation of muscle-building genes (67). Herein, the significant reduction in abdominal fat and lipid and cholesterol contents of breast and thigh muscles further highlights the lipid-lowering potential of Nio/Epicatechin. This effect may be attributed to its ability to inhibit lipogenesis by downregulating genes such as ACC and FAS, especially in broilers fed Nio/Epicatechin at the level of 300 mg/kg, while simultaneously promoting fatty acid oxidation through activation of AMP-activated protein kinase (AMPK), as previously documented (11). Similarly, upon activation, AMPK phosphorylates and inactivates ACC and suppresses the expression of FAS, thereby reducing the synthesis of new fatty acids and stimulating lipid catabolism (67). Moreover, studies in mammalian models have demonstrated that epicatechin enhances mitochondrial fatty acid oxidation, lowers hepatic triglyceride accumulation, and improves plasma lipid profiles through modulation of transcription factors including peroxisome proliferator-activated receptor alpha and sterol regulatory element-binding protein-1c (60). This dual regulation enhances fatty acid β-oxidation while suppressing triglyceride synthesis, resulting in a more favorable lipid profile in broiler meat in the current work. Taken together, the growth performance results in Table 3 and the carcass/proximate composition results of Table 5 appear mechanistically interconnected rather than independent outcomes. Specifically, the upregulation of *MyoD*, *MyoG,* and *mTOR* together with the downregulation of the *MSTN* gene (Figure 5) is proposed to drive the enhanced whole-body growth efficiency reflected in the higher BW, BWG, and lower FCR values of Table 3, while the concurrent downregulation of *ACC* and *FAS* and the associated AMPK-mediated stimulation of fatty acid oxidation redirect available nutrients away from adipose deposition and toward lean tissue accretion. This shared nutrient-partitioning mechanism is consistent with the higher breast and thigh yield percentages and the lower abdominal fat percentage observed in Table 5, linking the whole-animal growth response to the tissue-level carcass composition changes. The resulting reduction in intramuscular fat and cholesterol, along with increased protein deposition, contributes to an overall improvement in meat nutritional quality and healthfulness (68). Similar outcomes have been reported for other polyphenolic compounds such as catechins, quercetin, and green tea extract, which are known to enhance lipid metabolism, antioxidant capacity, and muscle development (69–71). These findings corroborate the hypothesis that nanotechnology can protect epicatechin, assisting its conveyance to target tissues and cells, along with maximizing its biological impacts regarding molecular aspects controlling myogenesis and lipogenesis (25).

The economic analysis observed in broilers fed the Nio/Epicatechin-containing diet can be primarily attributed to improvements in feed efficiency, growth rate, and nutrient utilization rather than direct reductions in diet formulation cost. In intensive broiler production systems, feed accounts for approximately 65–70% of total production costs; therefore, even modest improvements in feed conversion ratio have a pronounced impact on overall profitability (72). By increasing digestive efficiency, reducing oxidative stress, and strengthening gut integrity, Nio/Epicatechin improves feed efficiency by lowering the quantity of feed needed per unit of body weight gain. The current study showed that broilers fed the Nio/Epicatechin300-enriched diet gained more weight while consuming less feed, which resulted in a notable decrease in feed costs per kilogram of live weight produced. Similar outcomes have been reported in poultry studies evaluating polyphenol-rich feed additives, where improved feed conversion translated directly into reduced feed expenditure and enhanced economic returns. Additionally, oxidative stress reduces nutrient availability for growth and increases maintenance energy needs. A key factor in the economic effect of Nio/Epicatechin is the enhanced antioxidant status. As epicatechin directs dietary energy and amino acids toward muscle development rather than cellular repair activities by reducing oxidative damage (54). Our findings reported that net profit margins were increased and production expenses per unit of output were decreased owing to enhanced feed utilization efficiency. Additionally, intestinal inflammation, which is known to diminish feed efficiency and raise feed costs due to reduced nutrient absorption, is reduced by epicatechin-mediated regulation of gut microbiota (73). By reducing endogenous nutrient losses and improving nutrient digestibility, a healthy gut environment increases growth efficiency without requiring more feed. Ultimately, these biological advancements resulted in higher market body weight and better carcass yield, which raises overall revenue. Although the inclusion of Nio/Epicatechin containing ingredients may slightly increase total variable costs, the resulting improvements in body weight gain and FCR more than compensate for these additional inputs. This is reflected in higher net profit, benefit–cost ratio, and profit index values observed in broilers fed the Nio/Epicatechin300 diet. In agreement, previous economic assessments of functional feed additives in broilers have consistently shown that performance-driven gains, particularly improvements in feed efficiency, are the primary drivers of profitability rather than reductions in feed price alone (72). Ultimately, the inclusion of Nio/Epicatechin, particularly at higher levels in broiler diets, enhances economic efficiency by lowering feed cost per unit of production, increasing net profit, and improving return on investment.

Oxidative stress can initiate inflammatory pathways that subsequently influence the expression of genes related to antioxidant capacity, contributing significantly to the physiological functioning of the gastrointestinal tract (74, 75). In turn, this leads to an increase in intestinal permeability and intestinal barrier dysfunction (76). Flavonoids, which are natural secondary metabolites, possess antioxidant (77) and anti-inflammatory effects (78) that trigger animal production efficiency via modulating oxidative stress, inflammation, and intestinal damage (79). Here, the precise mechanism underlying the Nio/Epicatechin-mediated promotion of intestinal integrity and immune response was demonstrated via higher expression of tight junction-related genes and enhanced intestinal barriers. Moreover, structural modification of these intestinal tight junction proteins due to elevated oxidative stress or inflammatory response resulted in downregulation of occludin and ZO-1 expression. Meanwhile, flavones can modulate these impairments via enhanced intestinal tight junction barrier function through the assembly and expression of their associated proteins (80, 81). Previous research has focused on understanding the role of phytoconstituents, particularly flavonoids, in modulating the inflammatory response and protecting gut barrier integrity (82). The protective role of Nio/Epicatechin, shown by higher expression of tight junction-related genes, stems from its ability to balance the cellular redox state associated with oxidative stress and dysfunction of barrier-forming tight junctions (83). Enhanced expression of zona occludin and JAM suggests that epicatechin improves intestinal barrier function, a crucial factor for maintaining gut health and nutrient absorption. Previous studies have shown that catechins and other polyphenols can upregulate tight-junction proteins and reduce intestinal permeability under oxidative stress (84, 85). Besides, the anti-inflammatory cytokines IL-10 and IL-4 play a vital role in directing the immune response by limiting cell damage during inflammation (86). Moreover, the higher mRNA expression of IL-4 and IL-10 detected in the current study supports the anti-inflammatory potential of Nio/Epicatechin, consistent with reports that denoted attenuation of inflammatory cytokine production via inhibition of the NF-κB pathway following polyphenol fortification (21, 71). Defensins are indispensable peptides for host defense mechanisms that protect against microbial invasion. Furthermore, AVBD1 and cathelicidin-2 have been shown to play a key role in orienting the migration of immature dendritic cells to the inflammation site (87). Here, increased expression of AvBD1 and cathelicidin-2 following dietary supplementation with Nio/Epicatechin indicated its protective role against oxidative stress that predisposes to diseases. Notably, niosomal encapsulation improved the intestinal immunomodulatory role of Nio/Epicatechin by enabling effective delivery and controlled dosing. We hypothesize that the intestinal immunomodulatory response to Nio/Epicatechin was enhanced by an effective route of delivery and controlled dosing achieved through niosomal encapsulation (25, 27, 32); however, this delivery-related mechanism was not directly measured in the present study and is proposed based on the broader nanoencapsulation literature (28–30, 53, 88) rather than demonstrated experimentally here. The observed increase in AVBD1 and cathelicidin-2 expression further indicates that epicatechin not only enhances antioxidant capacity but also strengthens innate immunity. These antimicrobial peptides are key components of the host’s first-line defense and are known to be upregulated by polyphenolic compounds with immunostimulatory properties (69, 70). Comparable outcomes were reported by researchers (88), who demonstrated that catechin supplementation boosted β-defensin and cathelicidin expression in broilers under heat stress. Chronic low-grade inflammation (89) is known to impair growth performance by reallocating nutrients toward immune responses rather than growth processes (54). By mitigating inflammatory stress, Nio/Epicatechin improves nutrient partitioning efficiency, thereby supporting higher body weight gain. Overall, the growth-promoting effects observed in broilers fed the Nio/Epicatechin300 can be mechanistically linked to its antioxidant, metabolic, and gut-protective properties. These mechanisms collectively provide a biologically sound explanation for the superior growth performance recorded for Nio/Epicatechin (90).

Fresh meat quality properties, including cooking, drip, and thaw losses, color, and texture, significantly impact consumer purchasing behavior and the potential for meat to be further processed (3, 91). The glycolytic pathway is essential for the postmortem conversion of muscle to meat, primarily because it leads to the production of lactic acid, a key player in muscle pH reduction. The rate and efficiency of glycolysis directly determine the extent of the pH drop across muscle tissues, which in turn influence enzymatic activities and the rate of rigor development. The breakdown of glycogen and the resultant lactic acid production are closely linked to several quality parameters of meat. The elevated pH0.5, pH12, and pH24 values recorded in Nio/Epicatechin-fed broilers suggested a slower postmortem glycolytic rate and reduced lactic acid formation, resulting in a higher ultimate pH. By mitigating oxidative stress and maintaining mitochondrial function, epicatechin likely preserves ATP availability, thereby slowing glycolysis and stabilizing muscle pH. These findings align with earlier reports by Ouyang et al. (92), who demonstrated that antioxidant supplementation delays pH decline by limiting oxidative damage and metabolic disruption in postmortem muscle. For instance, an optimal rate of pH decline can prevent muscle protein denaturation, which is crucial for maintaining the meat’s water-holding capacity and texture. Conversely, a rapid or uneven pH decline can lead to PSE-like meat, characterized by a pale color and a watery, soft texture that is generally rejected by consumers. A higher ultimate pH directly correlates with improved water-holding capacity, as proteins remain more soluble and retain their capacity to bind water. Within the scope of this study, the improvements observed in meat quality attributes, including increased pH values and reductions in cooking, drip, and thaw losses following dietary supplementation with epicatechin, were remarkable in its niosomal formulation, which boosted its antioxidant and membrane-stabilizing properties. Consequently, meat from Nio/Epicatechin -supplemented broilers exhibited significantly lower cooking, drip, and thaw losses. These outcomes are consistent with findings by Shah (93), who reported similar improvements in meat quality in broilers supplemented with catechins and other polyphenols. These marked reductions in cooking, drip, and thaw losses may be partially attributed to upregulation of antioxidant defense genes including SOD-1, GSH-Px, and catalase, following feeding on Nio/Epicatechin, which neutralize reactive oxygen species and prevent oxidative modification of myofibrillar proteins (11). This protection maintained the structural integrity of muscle fibers and cellular membranes, reducing moisture loss and preserving tenderness, thereby improving meat quality.

Regarding meat color, the reduction in lightness (L)* and increase in redness (a)* values observed in this study indicated an improved pigment stability and reduced myoglobin oxidation. Epicatechin appears to inhibit the conversion of oxymyoglobin (Fe^2+^) to metmyoglobin (Fe^3+^), thus maintaining the desirable red coloration of fresh meat. Similar color-stabilizing effects of catechin-rich extracts were reported previously (68, 94), where enhanced redness and reduced lightness were noticed in broiler meat treated with polyphenolic antioxidants. The absence of significant changes in b* (yellowness) suggested that Nio/Epicatechin selectively stabilizes heme pigments without influencing carotenoid-based color components. At the molecular level, these protective effects may involve activation of the nuclear factor erythroid 2–related factor 2 (Nrf2)–ARE signaling pathway, which induces phase II antioxidant enzymes such as HO-1 and NQO1, which agrees with a previous report (10). This pathway enhances cellular resilience against oxidative stress, maintaining myoglobin in its reduced ferrous state and preventing pigment degradation. Additionally, epicatechin’s suppression of lipid peroxidation limits the formation of reactive aldehydes such as MDA and 4-HNE, which can interact with myoglobin and accelerate color deterioration (67). Comparable findings have been reported with dietary fortification using green tea catechins, which improved meat color stability and quality by reducing oxidative damage (10), and with quercetin or resveratrol, which can enhance pH and reduce lipid oxidation in broiler meat (95). Collectively, these findings confirmed the boosted antioxidant and metabolic modulator functions of Nio/Epicatechin that enhanced postmortem muscle stability, reduced oxidative protein and lipid damage, and thereby improved meat quality, color, and shelf life. We propose that these potential mechanistic actions may be partly ascribed to epicatechin nano-formulation-controlled delivery (96, 97), which is hypothesized to augment its release and cellular uptake and thus its efficacy (25, 27, 98); however, controlled release and cellular uptake were not directly assessed in the present study, and this remains a hypothesis supported by the broader nanoencapsulation literature (88, 99–101) rather than a mechanism demonstrated experimentally here. Herein, the significant upregulation of SOD-1, GPSH-Px, catalase, HO-1, and NQO1 in broilers supplemented with Nio/Epicatechin reflected a strong activation of the endogenous antioxidant defense system. This upregulation suggests that Nio/Epicatechin enhances cellular protection mechanisms by modulating redox-sensitive pathways, particularly the Nrf2–antioxidant response element signaling cascade. Activation of Nrf2 promotes the transcription of cytoprotective enzymes, such as HO-1 and NQO1, which play essential roles in maintaining redox homeostasis and preventing oxidative injury (60). These results align with previous reports where catechin-rich diets enhanced oxidative stability, reduced MDA accumulation, and extended meat shelf life (69, 70). Similar findings have been reported in broilers supplemented with other flavonoids, including quercetin, and grape seed polyphenols, which improved oxidative status through Nrf2 activation (54, 102). The improvement in muscle oxidative stability and carcass quality observed in this study can be attributed to these molecular adaptations. Upregulation of key antioxidant genes likely protected muscle cells against lipid peroxidation and protein oxidation, preserving membrane integrity and supporting muscle accretion.

Epicatechin, a member of the catechin family, acts as an effective free radical scavenger that protects muscle proteins and lipids from oxidative deterioration, thereby enhancing the physicochemical and sensory quality of meat (103). Epicatechin appears to function through a dual mechanism, acting as both a direct antioxidant, capable of scavenging free radicals, and an indirect modulator that induces the expression of antioxidant and cytoprotective genes (60). This dual mechanism, combining lipid metabolism regulation and antioxidant defense, ensures the preservation of health-promoting fatty acids and minimizes oxidative degradation during storage. However, the magnitude of these effects depends on factors such as dosage, bioavailability, stability, and controlled release attributed to its incorporation into niosomes (30). Likewise, incorporation of epicatechin into a nano form in the current research enhanced this meat’s free radical scavenging activity during the 30- and 90-day frozen storage period. Taken together, these results highlight the role of epicatechin as a natural metabolic modulator that promotes fatty acid oxidation, suppresses lipogenesis, and protects lipids from oxidative deterioration over long frozen storage periods. Herein, the observed reduction in MDA and total volatile aldehydes in broiler breast meat indicated that dietary Nio/Epicatechin effectively attenuates lipid oxidation during refrigerated storage. MDA, a principal secondary product of lipid peroxidation, is widely recognized as a reliable indicator of oxidative damage and meat spoilage. The significant decline in MDA levels in birds fed Nio/Epicatechin -supplemented diets reflects enhanced antioxidant defense and stabilization of muscle lipids. Previous research reported comparable results (21), where reduced MDA formation in broiler meat following supplementation with catechin-rich plant extracts was observed, attributed to their potent free radical–scavenging and metal-chelating abilities. Epicatechin, a flavan-3-ol containing multiple hydroxyl groups, can donate hydrogen atoms or electrons to neutralize lipid peroxyl radicals, thereby interrupting the propagation phase of lipid oxidation. Additionally, catechins chelate pro-oxidant metal ions such as Fe^2+^ and Cu^2+^, which catalyze lipid peroxidation through Fenton-type reactions (104). This dual antioxidant mechanism radical scavenging and metal chelation—contributes to the preservation of lipid stability in muscle tissues. Moreover, epicatechin supports the maintenance of endogenous antioxidant systems, including SOD, catalase, and GSH-Px, thereby strengthening the cellular defense network against oxidative stress (105). The current findings on concurrent reductions in cholesterol oxidation products further highlight the role of Nio/Epicatechin in suppressing cholesterol autoxidation. Cholesterol oxidation, which is typically accelerated during storage and thermal processing, generates cytotoxic compounds such as 7-ketocholesterol and 7-hydroxycholesterol, both of which impair meat quality, nutritional value, and consumer safety. The observed inhibition of cholesterol oxidation product formation in Nio/Epicatechin-fed broilers aligns with recent findings (69, 70), where dietary polyphenols effectively reduced cholesterol oxidation and extended meat shelf life. Similarly, researchers (30) demonstrated that green tea catechins and grape seed polyphenols minimized lipid and cholesterol oxidation in stored chicken and pork meat, respectively. The observed reduction in cholesterol oxidation product contents is especially important from a nutritional standpoint, as it is associated with reduced inflammation, improved cardiovascular health, and overall better metabolic outcomes in humans (21, 69, 70, 106). The persistence of measurable antioxidant capacity (DPPH, ABTS and T-AOC) despite the progressive decline in total phenolic and flavonoid content over the course of frozen storage may reflect the concurrent upregulation of endogenous antioxidant enzyme genes (*SOD-1*, catalase, *GSH-Px*, *HO-1* and *NQO1*), which continue to confer cellular free-radical scavenging and reducing capacity independently of the exogenous phenolic pool. Additional contributions may arise from non-phenolic reducing metabolites generated during storage and from synergistic interactions among the residual bioactive compounds, both of which can sustain measurable antioxidant activity even as the parent phenolic and flavonoid content is progressively consumed through ongoing radical-scavenging reactions. The vertices of the fitted second-order models cluster in the upper part of the range examined, but they differ in whether they fall inside or outside that range and must be interpreted accordingly. The vertices estimated for body weight gain (247 mg/kg diet), drip loss (277 mg/kg diet), total phenolic content (256 mg/kg diet) and DPPH radical-scavenging activity (279 mg/kg diet) after 90 days of frozen storage lie inside the experimental range and are interpolated predicted optima, whereas those estimated for breast crude protein (307 mg/kg diet), T-AOC (315 mg/kg diet), breast redness (349 mg/kg diet) and abdominal fat (351 mg/kg diet) exceed the highest level fed and are extrapolations that were not experimentally validated. Taken together with the observation that FCR and the antioxidant indices were still improving at the highest level tested, these estimates support an inclusion level close to 300 mg/kg diet, the highest level actually evaluated and the best-performing one for feed conversion, while indicating that any further benefit above this level remains a model-based projection that would require direct testing of higher inclusion rates before it could be recommended. Collectively, these results suggest that Nio/Epicatechin supplementation up to 300 mg/kg markedly enhanced the oxidative stability of broiler meat by inhibiting both lipid peroxidation and cholesterol oxidation pathways. This protective effect appears to preserve the nutritional value of the meat and to contribute to improved oxidative storage stability through the reduced formation of lipid and cholesterol oxidation products.

## 5 Conclusion

Collectively, the results of this study established that dietary supplementation with Nio/epicatechin exerted advantageous properties on broiler health, physiological performance, and meat quality. The results indicated that Nio/epicatechin enhanced antioxidant defense systems, boosted immune competence, and reinforced intestinal integrity. These molecular and physiological changes contribute to superior oxidative stability, improved gut functionality, and better overall productivity in broiler chickens. Furthermore, Nio/epicatechin supplementation promoted muscle development, increased carcass yield, and reduced fat deposition, thereby improving the nutritional value and quality of broiler meat, extending meat shelf life, and refining consumer acceptability. From a practical perspective, the findings suggest that Nio/epicatechin-based feeding strategies could be economically reasonable and effectively implemented in commercial broiler production. Overall, Nio/epicatechin represents a promising natural bioactive compound capable of enhancing poultry performance, health status and meat quality highlighting its potential as a supportable alternative to conventional growth-promoting additives.

## Data Availability

The original contributions presented in the study are included in the article/Supplementary material, further inquiries can be directed to the corresponding authors.
